# *Neisseria meningitidis* regulates P-glycoprotein transporter activity in brain endothelial cells via sphingosine 1–phosphate receptor 1

**DOI:** 10.1186/s12987-025-00687-0

**Published:** 2025-07-22

**Authors:** Fatemeh Nosratabadi, Leo M. Endres, Fabian Schumacher, Heike Claus, Burkhard Kleuser, Brandon J. Kim, Alexandra Schubert-Unkmeir

**Affiliations:** 1 Institute for Hygiene and Microbiology, University of Würzburg, Josef-Schneider-Strasse 2, 97080 Würzburg, Germany; 2 Institute of Pharmacy, Freie Universität Berlin, Berlin, Germany; 3 Department of Biological Sciences, University of Texas at Dallas, Richardson, TX USA

**Keywords:** *Neisseria meningitidis*, Brain endothelial cells, Efflux transporter, P-glycoprotein, Breast cancer resistance protein, Sphingosine 1-phosphate receptor 1

## Abstract

**Background:**

The brain endothelial cells (BECs) are essential for protecting the central nervous system (CNS) from xenobiotics and pathogens, including *Neisseria meningitidis*, while maintaining CNS homeostasis through tight junction (TJ) proteins and specialized transporters. Among these, multidrug resistance (MDR) transporters such as P-glycoprotein (P-gp) and breast cancer resistance protein (BCRP) are pivotal in restricting the entry of neurotoxic substances. Although the impact of *N. meningitidis* infection on BBB TJ is well-documented, its effect on MDR transporters remains largely unexplored.

**Methods:**

We employed induced pluripotent stem cell-derived brain-like endothelial cells (iBECs) as an in vitro BECs model due to their human-like morphology and expression of junctional proteins and MDR transporters. iBECs were exposed to various *N. meningitidis* strains, isogenic mutants, heat-inactivated bacteria, conditioned media, or purified capsule polysaccharide (CPS). P-gp and BCRP activities were assessed using intracellular accumulation assays with Rhodamine 123 and Chlorin e6, respectively, in the presence of P-gp inhibitors cyclosporin A and PSC833 and BCRP inhibitor Ko143. Gene expression and protein levels were determined by qPCR and western blotting, and sphingolipid quantification was performed via liquid chromatography tandem-mass spectrometry (LC-MS/MS).

**Results:**

Infection of iBECs with *N. meningitidis* inhibited P-gp activity, whereas BCRP activity remained unaffected. P-gp inhibition occurred without changes in gene expression or protein abundance. Cells infected with *N. meningitidis* showed reduced efficacy of P-gp inhibitors, an effect not seen with the BCRP inhibitor Ko143. *N. meningitidis* CPS was identified as a key factor in modulating P-gp activity. Notably, the inhibitory effect of *N. meningitidis* on P-gp activity was blocked by a specific sphingosine 1-phosphate receptor 1 (S1PR_1_) antagonist as well as by sphingosine kinase inhibitors, revealing a mechanistic link between S1PR_1_ signaling and P-gp modulation during infection. Furthermore, S1PR_1_ was upregulated in infected iBECs. Although LC-MS/MS measurement showed no increase in S1P levels in infected cells compared to uninfected controls, these findings suggest a crucial role for S1PR_1_ signaling in mediating the observed effects.

**Conclusions:**

These findings demonstrate that *N. meningitidis* infection impairs P-gp function through S1PR_1_-dependent pathways, suggesting that targeting this signaling cascade may offer a novel therapeutic strategy to preserve BBB integrity during bacterial infections.

**Supplementary Information:**

The online version contains supplementary material available at 10.1186/s12987-025-00687-0.

## Background

The blood-brain barrier (BBB), along with the meningeal blood-cerebrospinal fluid barrier (mBCSFB), is crucial for maintaining central nervous system (CNS) homeostasis and protecting the brain from harmful substances in the circulation [1, 2]. These barriers are primarily composed of brain endothelial cells (BECs) that exhibit unique phenotypes characterized by low rate of transcytosis, complex tight junctions (TJs) between the cells, and specialized efflux transporters, including P-glycoprotein (P-gp) and breast cancer resistance protein (BCRP) [1]. The non-fenestrated nature of BECs and their TJs effectively limit paracellular passage into the brain. Highly lipophilic solutes are expected to traverse biological BEC barriers readily, however, their passage is often impeded by the active efflux mechanisms of adenosine triphosphate (ATP)-binding cassette (ABC) transporters expressed in the brain endothelium [3]. These transporters are abundantly expressed at the luminal surface of the BECs and prevent their substrates from penetrating the brain [4–7]. Among the most prominent multidrug resistance (MDR) transporters at the BBB are P-gp (170 kDa, encoded by *ABCB1*) and BCRP (65–80 kDa, encoded by *ABCG2*) [8, 9]. By actively restricting the accumulation of various endogenous and exogenous compounds in the brain, these transporters serve as a crucial defense mechanism that limits the entry of drugs and xenobiotics from the circulation [10–12]. Several studies have demonstrated that inflammatory processes, such as those associated with neurological disorders including multiple sclerosis, Alzheimer’s and Parkinson’s diseases, as well as infections, can significantly impact on the function of these transporters [10, 13–16]. Under such pathological conditions, compensatory mechanisms often emerge, wherein a decrease in the expression or activity of one transporter is accompanied by an increase in the expression and/or activity of the other [13, 17–19]. Specific intracellular signaling pathways have been implicated in the regulation of transporter activity. For instance, estrogen signaling has been shown to modulate BCRP activity, whereas sphingolipid signaling influences P-gp function in the context of cancer [20–22]. These findings highlight the dynamic and adaptive nature of transporter regulation under pathological conditions.

*Neisseria meningitidis*, commonly referred to as meningococcus, is a Gram-negative diplococcal bacterium that colonizes the human nasopharynx as a commensal organism [23]. However, in rare instances, it becomes highly pathogenic, breaching the nasopharyngeal epithelial barrier to enter the bloodstream, where it causes severe septicemia or crosses the BECs of the mBCSFB to induce meningitis [24]. The pathogenic nature is based on key bacterial virulence factors, including the polysaccharide capsule, the lipooligosaccharide, type IV pili (Tfp), and the OpcA outer membrane protein, which facilitate bacterial adhesion and invasion of host cells [25–28]. Upon binding to receptors on BECs, *N. meningitidis* triggers intracellular signaling cascades, leading to actin cytoskeleton reorganization and alterations in the lipid composition of the host cell membrane, which promote bacterial uptake [24, 29–31].

The interaction of *N. meningitidis* with BECs and its traversal across the endothelial barrier of the mBCSFB represent a key step in the pathogenesis of meningococcal meningitis. Tfp of *N. meningitidis* have been shown to mediate adhesion to BECs by engaging CD147, a transmembrane glycoprotein, also known as basigin [32, 33]. CD147 forms a complex with the β2-adrenoceptor (β2AR), and Tfp binding triggers β2AR-biased signaling, leading to β-arrestin recruitment and localized cytoskeletal remodeling beneath the bacterial microcolonies [34]. Concurrently, β2AR signaling activates Src kinases, adherens junction proteins, TJ proteins, and the Par3/Par6/PKCζ complex to form a cortical plaque resulting in failure of the TJ between BECs, compromising endothelial barrier integrity and facilitating bacterial paracellular traversal [34]. In addition, proteolytic cleavage of occludin, which is mediated by matrix metalloproteinases, also leads to a weakening of barrier integrity [35]. While the reorganization and disruption of TJs and the alteration of endothelial barrier integrity during infection have been documented, the potential impact of *N. meningitidis* infection on MDR transporters in BECs has not been investigated.

Our findings demonstrated that *N. meningitidis* infection specifically inhibited P-gp activity, while BCRP activity remained unaffected. Further analysis revealed that the polysaccharide capsule of *N. meningitidis* is essential for this inhibition, which is mediated downstream of sphingolipid signaling involving sphingosine 1-phosphate receptor 1 (S1PR_1_). These changes compromise the protective functions provided by P-gp at the mBCSFB, underscoring a previously unrecognized mechanism by which *N. meningitidis* disrupts mBCSFB integrity.

## Methods

### Cell culture and brain-like endothelial cell differentiation

Human induced pluripotent stem cells (iPSCs) line IMR90-4 (WiCell) was differentiated into iPSC-derived brain-like endothelial cells (iBECs) as previously described [36–38]. Briefly, iPSCs were seeded on Matrigel (Corning) coated 6-well plates (Sarstedt) in StemFlex medium (Gibco) and passaged upon reaching 60–80% confluence. To ensure the quality of the cultures, iPSC colonies were characterized by immunofluorescence staining for pluripotency-associated markers, including OCT3/4, NANOG and TRA1-81 (Fig. S1A). Mycoplasma testing was periodically conducted using a PCR-based kit (Merck, MP0035). Differentiation of iPSCs into a mixed population of iBECs and neural progenitor cells was initiated by switching to an unconditioned medium (UM; DMEM/F-12 (Gibco), 20% knockout serum replacement (Gibco), 1% minimal essential medium-nonessential amino acids (Gibco), 0.5% GlutaMAX (Gibco), and 0.07% beta-mercaptoethanol (Sigma-Aldrich)) for six days with daily media changes (day 0–5). iBEC population was expanded by changing the medium to a human endothelial serum-free medium (hESFM, Thermo Fisher) containing 1% B27, 20 ng/ml basic fibroblast growth factor (bFGF), and 10 µM retinoic acid (RA) (referring to as EC++), from day 6 without changing medium for 48 h. To purify the iBECs, on day 8 the mixed cultures were collected with accutase (Merck) and were subcultured onto collagen IV and fibronectin-coated plates or transwell inserts in EC + + for 24 h. By day 9 a pure monolayer of iBECs formed and the medium was switched to hESFM supplemented with 1% B27 (referred to as EC–; endothelial cell assay medium). The quality of differentiated iBECs was assessed by measurements of the transendothelial electrical resistance (TEER) and immunofluorescence staining of BEC markers (Figs. S1B and C).

### Meningococcal strains

The bacterial strains used in this study are listed in Table 1. All strains were grown overnight on Columbia agar with 5% sheep blood (bioMérieux) at 37^o^C and 5% CO_2_. The following day, bacteria were routinely cultured in liquid protease peptone medium (PPM) freshly supplemented with 1% Kelloggs reagent [39], 10 nM MgCl_2_ (Carl Roth) and 10 nM NaHCO_3_ (Merck) (referred to as PPM++) at 37^o^C and 200 rpm for 90 min.

**Table 1:** Bacterial strains used in this study

Bacteria/mutants	Other information	Reference
*N.m.* MC58 *N.m.* MC58Δ*csb* *N.m.* MC58Δ*opcA* *N.m.* MC58Δ*pilE* *N.m.* H44/76 *N.m.* H44/76Δ*Ipx* *N.m.* WUE2120	WT, serogroup B, ST-32/[ST-74], kindly provided by E.R. Moxon Isogenic unencapsulated mutant Isogenic OpcA-deficient mutant Isogenic non-piliated mutant WT, serogroup B, ST-32 Isogenic LOS-deficient mutant WT, serogroup C, ST-11	[40] [41] [41] [31] [42] [43] [44]

### Infection assays

On the day of infection (day 10 of differentiation), fresh EC– was added to the cells at least 1 h before starting the infection. The OD of bacteria grown 90 min at 37^o^C in PPM + + was measured at 600 nm. 1 ml of bacteria was spun down at 3,320 x g for 5 min and resuspended in 1 ml of EC–. For heat-inactivation (HI) of the bacteria, 1 ml of the bacterial pre-culture was heated at 95 °C for 20 min. After heat treatment, the culture was spun down, and the pellet was resuspended in 1 ml fresh EC– medium. *N. meningitidis-*conditioned medium (CM) was generated by growing strain MC58 in endothelial cell assay medium (EC–) for 90 min, after centrifugation at 3,320 x g for 10 min, the supernatant was collected and filtered using a 0.2 μm pore vacuum filter. To verify the absence of any bacterial contamination 100 µl of the filtered supernatant was plated on blood agar and incubated overnight at 37^o^C, 5% CO_2_. For all experiments, two wells were used to determine the cell number and multiplicity of infection (MOI) of 100 was used for all experiments. For experiments involving HI bacteria or CM, the MOI was adjusted by measuring the bacterial OD_600_ prior to inactivation and filtration. The infection was carried out at 37^o^C and 5% CO_2_.

### P-gp and BCRP activity assay

P-gp and BCRP activity was assessed by measuring the intracellular accumulation of Rhodamine 123 (R123, 10 µM, Sigma-Aldrich) and Chlorin e6 (Ce6, 1 µM, Santa Cruz), respectively [13, 14, 16]. The uptake of R123 and Ce6 by P-gp or BCRP was confirmed under exposure to their specific inhibitors, Valspodar (PSC833) or Cyclosporin A (CsA) and Ko143, respectively [13, 16, 45, 46]. Briefly, on day 8 of differentiation, the cells were seeded at a density of 500,000 cells per well in a 24-well plate. On day 10, cells were infected with wild-type (WT) strain MC58 or isogenic mutants at an MOI of 100 for 4 or 6 h or treated with a volume of HI *N. meningitidis* MC58 or MC58- CM equivalent to an MOI of 100 for 4 h. Purified capsular polysaccharide (CPS), dissolved in 1 x PBS, was added to the cells at varying concentrations for 4 h, the final PBS concentration in each well remained below 0.03 x PBS. Preliminary experiments indicated that PBS concentrations ≤ 0.03 x PBS had no impact on transporter activity (Fig. S2A). After infection, cells were washed with pre-warmed Hank’s Balanced Salt Solution (HBSS) (Gibco) and incubated with or without inhibitor (10 µM PSC833, 10 µM CsA, 10 µM Ko143) for 1 h at 37^o^C + 5% CO_2_ [16]. Subsequently, the cells were incubated for 2 h with the specific substrate for P-gp or BCRP, with or without inhibitors. After incubation, the cells were washed twice with ice-cold 1 x PBS, lysed with 200 µl radioimmunoprecipitation assay buffer (RIPA buffer; 1 M Tris-HCl pH 6.8, 2% SDS, 10% glycerol, containing 1% protease and phosphatase inhibitor cocktail from Thermo Scientific) per well, and placed on a rocker for 10–15 min at room temperature (RT), protected from light. The fluorescent intensities of R123 and Ce6 were measured using the SpectraMax iD3 microplate reader (Molecular devices) at excitation/emission wavelengths of 485/530 nm and 407/667 nm, respectively [13, 16]. Fluorescent values were normalized to protein concentration and the results were expressed as a percentage of baseline controls. In additional activity experiments, S1P (diluted in 4 mg/ml fat-free BSA in 1 x PBS) was added during the last 20 min of substrate incubation, S1PR_1_ antagonist (W146; diluted in DMSO) and Sphingosine Kinase 1 and 2 inhibitors (PF543; SphK1 inhibitor, SLM6031434; SphK2 inhibitor, both diluted in DMSO), prepared in cell culture assay medium, were added 1 h before infection and maintained throughout the assay. In parallel, vehicle controls were tested to account for any effects of the diluents.

### Purification of *Neisseria meningitidis* capsular polysaccharide

To purify CPS from *N. meningitidis* strain MC58, bacteria were cultured overnight on 50 Columbia agar plates with 5% sheep blood (bioMerieux) at 37^o^C and 5% CO_2_. The bacteria were harvested and suspended in 900 ml 1 x PBS, resulting in an OD_600_ of approximately 0.6–0.7. CPS were released overnight at 4^o^C by lysing with 10% of Cetavlon (hexadecyltrimethylammonium bromide, Sigma-Aldrich), added to a final concentration of 1.0%. The bactericidal efficacy of Cetavlon was confirmed by plating 100 µl suspension on Columbia agar plates. The precipitate and bacterial debris were collected by centrifugation at 13,700 x g for 30 min at 4^o^C using a GS3 rotor in a Sorvall RC-5B Refrigerated Superspeed Centrifuge. The pellet was resuspended in 15 ml of distilled water and mixed with an equal volume of 2 M CaCl_2_, followed by stirring for 1 h at 4^o^C to dissociate CPS-Cetavlon complexes. To remove nucleic acids, cellular fragments, and large proteins, 25% ethanol (EtOH) was added, and the mixture was incubated for 2 h at 4 °C. Subsequent centrifugation steps were performed at 4 °C in Teflon tubes in a HB-6 rotor in a Sorvall RC-5B Refrigerated Superspeed Centrifuge. After centrifuging at 27,600 x g for 20 min, the supernatant was adjusted to 80% EtOH to precipitate the polysaccharides, followed by another round of centrifugation. The pellet was then resuspended in distilled water and underwent additional processing with 2 M CaCl_2_ and 25% EtOH, with the mixture stirred overnight at 4^o^C. After centrifugation at 27,600 x g for 20 min, the pellet was washed three times with acetone and twice with diethyl ether. It was dried at RT for 45 min and subsequently resuspended in 15 ml of 10% Na-acetate, stirring with a magnetic stir bar in a teflon tube at RT for about 1 h—following, an equal volume of phenol (containing 2.1% Na-acetate) was added. The suspension was allowed to stir rapidly in an ice bath for 30 min to enable phase separation. The mixture was centrifuged at 27,600 x g for 20 min to separate the phases. The clear upper phase, containing the CPS, was transferred to a glass bottle and precipitated with 80% EtOH, followed by centrifugation at 5,883 x g for 10 min. The residue was dissolved in 12 ml of 10% Na-acetate, and the Na-acetate-phenol precipitation cycle was repeated. The final EtOH precipitation was allowed to proceed overnight at 4^o^C. The pellet was dissolved in 10 ml distilled water and subjected to ultracentrifuge at 100,000 x g for 5 h at 4^o^C using a Ti41 rotor in a Thermo SCIENTIFIC SORVALL wX + ULTRA SERIES ultracentrifuge to remove lipooligosaccharides. The supernatant was supplied with EtOH to the final concentration of 80% and centrifuged at 5,883 x g in a HB-6 rotor in a Sorvall RC-5B Refrigerated Superspeed Centrifuge for 10 min. The final precipitate was washed three times with EtOH and two times with acetone, increasing centrifugation times at 5,833 x g between each step. After drying the pellet overnight at RT, it was dissolved in 150 µl of 1 x PBS and stored at -20 °C for further analysis. The quantity and purity of the purified CPS were evaluated using enzyme-linked immunosorbent assay (ELISA).

### ELISA for quantitative determination of CPS

Identification and quantification of the purified CPS were performed using ELISA. 96-well microtiter plates were pre-treated with 50 µl poly-D-lysine (25 ng/ml 1 x PBS) and incubated 30 min at RT. Antigen attachment, either bacterial suspensions (2 × 10^8^ bacteria in 1 ml 1 x PBS), or purified CPS diluted in 1 x PBS, was carried out by incubating 20 µl of each sample per well for 1 h at 37^o^C. *N. meningitidis* MC58 and isogenic unencapsulated mutant strain MC58*∆csb* were used as controls. For bacteria, an additional fixation step was performed using 100 µl of 0.05% glutaraldehyde in 1 x PBS for 10 min. Subsequently, the wells were washed 3 times with 1 x PBS, and blocking was done for 30 min with 150 ml 1% BSA in 1 x PBS. 20 µl of primary antibody against *N. meningitidis* CPS serogroup B; (mAb735 from mouse, diluted 1:4,000 in 1% BSA) was added per well [47]. The plates were incubated for 1 h at RT. The secondary antibody anti-mouse IgG + M (H + L) conjugated to horseradish peroxidase (HRP) (1:2,500 in 1% BSA) was allowed to incubate for 45 min. Following incubation, the plates were washed twice, and 20 µl per well of 2,2’-casino-bis (3-ethylbenzothiazoline-6-sulfonic acid) (ABTS; 1 mg/ml in ABTS buffer (Roche)) was added for 10 min. The absorbance was measured at 414 nm using a MULTISKAN EX spectrophotometer (ThermoScientific). Colominic acid sodium salt from *Escherichia coli* (Sigma-Aldrich, Cat. No. K0923) was serially diluted in 1 × PBS and used as a standard to estimate the concentration of the CPS preparation.

The same ELISA methodology was employed to assess the presence of *N. meningitidis* CPS in HI and CM samples. The WT *N. meningitidis* MC58 strain and its encapsulation mutant were cultured in PPM + + medium for 90 min. After incubation, bacteria were collected by centrifugation, the pellet was resuspended in 1 x PBS. The OD_600_ was adjusted to 1 for all samples. For HI, 1 ml of the WT culture with OD_600_ = 1 was subjected to heat treatment at 95 °C for 20 min. Subsequently, bacterial samples (live and HI) were centrifuged, and the pellet was resuspended in 1 ml 1 x PBS to ensure uniform handling across all samples. For CM, bacteria were cultured in the cell culture assay medium. After incubation, the entire culture spun down, and the supernatant was filtered to remove bacterial cells. A total of 20 µl from each sample was transferred to pre-coated microtiter plates. All samples were subsequently fixed with 0.05% glutaraldehyde in 1 x PBS for 10 min. The absorbance at 414 nm was measured to evaluate the presence of *N. meningitidis* CPS in each sample. Results are shown in Supplementary Figure S2B.

### Cell viability assay

The cytotoxicity effects of agents on iBECs were determined by measuring lactate dehydrogenase (LDH) levels in the medium using the LDH cytotoxicity detection kit (Roche #71804100), following the manufacturer’s instructions and data are presented in Supplementary Figure S3.

### Bacterial growth curves

Bacterial growth kinetics in EC– were performed as described recently [31]. OD_600_ was measured every 1 h over a period of 360 min and data are shown in Supplementary Figure S4. Additionally, the growth of WT MC58 and MC58Dcsb was compared by plating 100 µl of bacterial suspension on blood agar every two hours following serial dilutions. Colony-forming units (CFU) were then quantified to assess any differences in bacterial load between the strains (Fig. S4D).

### Bactericidal activity test of inhibitors/receptor antagonist

Bactericidal activity of sphingosine kinase inhibitors (10 µM PF543 (SphK1 inhibitor), 1 µM SLM6031434 (SphK2 inhibitor)), and the S1PR_1_ receptor antagonist (1 µM W146) was assessed in EC-. Bacteria were pre-cultured in 10 ml of PPM + + for 90 min, adjusted to OD_600_ = 1, centrifuged, and resuspended in 1 ml of EC–. The suspension was diluted to OD_600_ = 0.1 in 10 ml of EC–, with either the test agent or vehicle control (DMSO). The bacterial inoculum was incubated with shaking at 200 rpm and 37 °C, and OD_600_ was measured every 30 min for 240 min. Data are shown in Supplementary Figure S5.

### Immunofluorescence staining of iPSC markers

iPSCs were seeded on Matrigel-coated 12-well plates at a density appropriate for their growth and cultured in StemFlex medium. After 3 days, when the cells reached approximately 70–80% confluence, the medium was changed daily, and staining was performed. The cells were washed with 1 x PBS and fixed with 4% paraformaldehyde (PFA) for 15 min. For OCT3/4 and NANOG staining, cells were permeabilized using 0.1% Triton X-100 in 1 x PBS for 15 min, followed by three washes with 1 x PBS (5 min each). The cells were then blocked with 3% BSA in sterile 1 x PBS for 30 min. The primary antibodies OCT3/4, NANOG, and TRA-1-81 were diluted in the blocking solution and incubated with the cells overnight at 4 °C. The following day, the cells were washed with 1 x PBS (3 times for 5 min). Secondary antibodies, Alexa 488 and Alexa 555 (1:200), were added in the blocking solution, and cells were incubated at room temperature for 60 min, and protected from light. After another round of washing with 1 x PBS (3 times for 5 min), DAPI (Invitrogen, #D1306) diluted 1:5,000 in 1 x PBS was added for 15 min at room temperature to stain the cell nuclei. The cells were then washed once more with 1 x PBS (5 min) and maintained in 1 x PBS for imaging. Fluorescent images were captured using an Eclipse Ti fluorescence microscope (Nikon) at 10 x magnification. Images are shown in Supplementary Figure S1A. The list of the antibodies used, and their dilution are given in Table 2.

### iBECs characterization using immunocytochemistry

Immunohistochemical analyses were performed as previously described to verify the phenotype of the iBECs model [36]. Briefly, iBECs monolayers were seeded onto 8-well ibidi slides (Cat. No. 80821). The wells were pre-coated with collagen IV and fibronectin. On day 10 of differentiation, cells were washed once with 1 x PBS and fixed for 15 min in ice-cold methanol at RT. Subsequently, the cells were washed three times and blocked for 1 h in a blocking solution consisting of 1 x PBS with 10% FCS at RT. Primary antibodies were diluted in the blocking solution and incubated with the cells overnight at 4^o^C. The following day, the cells were rinsed and secondary antibodies, Alexa Flour 488-conjugated goat anti-mouse or Alexa Flour 555-conjugated donkey anti-rabbit, were applied for 1 h at RT. The cells were washed three times with 1 x PBS and nuclei were stained with DAPI as described above, followed by a final wash step. Staining was visualized on an Eclipse Ti2 confocal microscope (Nikon) at 100 X magnification. Control samples were treated solely with secondary antibodies to detect any non-specific binding. NIS Elements image software version 5.02 (Nikon) was utilized for image processing, with consistent channel settings and color correction maintained in all images. Images are shown in Supplementary Figure S1D. The list of the antibodies used, and their dilution are given in Table 2.

**Table 2:** Antibodies used for Immunofluorescence

Target antigen	Dilution	Species and clonal information	Vendor and catalogue number
VE-Cadherin^a^	1:25	Ms, clone BV9	Santa Cruz, sc-52,751
ZO-1^b^	1:100	Ms, clone ZO1-1A12	Invitrogen, 33-9100
CD31^c^	1:200	Rb, polyclonal, ab32457	Abcam, ab32457
Occludin^a^	1:200	Ms, clone OC-3F10	Invitrogen, 33-1500
Claudin-5^a^	1:50	Ms, clone 4C3C2	Invitrogen, 35-2500
P-glycoprotein^a^	1:50	Ms, clone F4	Invitrogen, MA5-13854
OCT3/4	1:500	Ms, monoclonal, C-10	Santa Cruz, sc-5279
NANOG^d^	1:200	Rb, monoclonal, clone D73G4	Cell signaling, 4903T
TRA-1-81^d^	1:1000	Ms, monoclonal	Cell signaling, 4745T
Wheat Germ Agglutinin (WGA)	5 µg/ml	Alexa Fluor Plus 405 conjugated	Invitrogen, W56132
Alexa Flour 488 goat anti-mouse	1:200	Goat, polyclonal	Invitrogen, A11001
Alexa Flour 555 donkey anti-rabbit	1:200	Donkey, polyclonal	Invitrogen, A31572
Alexa Fluor 555 donkey anti-mouse	1:200	Donkey, polyclonal	Invitrogen, A31570

^a^Stebbins et al. 2016 [36], ^b^Kim et al. 2017 [48], ^c^Endres et al. 2022 [49]. ^d^Chen et al. 2024 [50]

### Immunoblotting

Proteins were extracted from infected iBECs using RIPA buffer (50 mM Tris-HCl pH 7.2, 150 mM NaCl, 5 mM EDTA, 1% Triton X-100, 24.1 mM sodium deoxycholate, 0.1% SDS, 50 mM sodium fluoride) supplemented with a protease and phosphatase inhibitor cocktail (Thermo Scientific™). Lysates were incubated on ice for 30 min, centrifuged at 12,000 × g for 15 min at 4 °C to remove debris, and the supernatants were quantified using the Pierce BCA Protein Assay Kit. Samples were stored at -20 °C. For immunoblotting, 15 µg of protein was mixed with 4 × SDS loading buffer, incubated at 70 °C for 10 min, separated on NuPAGE 4–12% Bis-Tris gels (Invitrogen), and transferred to nitrocellulose membranes (Merck). Membranes were blocked in 5% skim milk in Tris-buffered saline with 0.1% Tween 20 (TBS-T), incubated overnight at 4 °C with the following primary antibodies: anti-P-glycoprotein (#MA5-13854, ThermoFisher, 1:1,000 in 5% skim milk), anti-BCRP (#42078, Cell Signaling, 1:1,000 in 5% BSA), and GAPDH antibody (#60004-1-IG, Proteintech, 1:50,000 in 5% skim milk). HRP-conjugated secondary antibodies (anti-rabbit or anti-mouse, 1:5,000) were applied, and detection was performed using enhanced chemiluminescence (ECL) substrates (BioRad). Imaging was performed using a BioRad ChemiDoc imager, and quantification was carried out using FIJI ImageJ software v1.54j, normalizing protein signals to GAPDH.

### RNA extraction and reverse transcription quantitative PCR (RT-qPCR)

Monolayers of iBECs were either left uninfected or were infected with *N. meningitidis* MC58. After infection, total RNA was extracted using a NucleoSpin RNA kit (Macherey Nagel) according to the manufacturer’s instructions and RT-qPCR was performed as described recently [49]. Primers used in this study were purchased from Sigma and are listed in Table 3. For all primers used in RT-qPCR, the annealing temperature was optimized. Primer efficiency and linearity were validated. Specificity was assessed through melt curve analysis and confirmed with agarose gel electrophoresis.

**Table 3:** Primers used for RT-qPCR

Gene	Forward sequence	Reverse sequence
*ABCB1* ^a^	GAAGAGATTGTGAGGGCAGC	CCACCAGAGAGCTGAGTTCC
*ABCG2* ^b^	TCAGGTCTGTTGGTCAATCTC	GTTTCCTGTTGCATTGAGTCC
18 S rRNA^c^	GTAACCCGTTGAACCCCATT	CCATCCAATCGGTAGTAGCG
*S1PR1* ^d^	CTCCGTGTTCAGTCTCCTCG	ATTGCTCCCGTTGTGGAGTT
*S1PR2* ^e^	CATCGTCATCCTCTGTTG	AGTGGAACTTGCTGTTTC
*S1PR3* ^d^	CGGCATCGCTTACAAGGTCAAC	GCCACGAACATACTGCCCTC

^a^Brandon et al. 2019 [16],^b^Lippmann et al. 2012 [37] ^c^Rho et al. 2010 [51], ^d^Fohmann et al. 2023 [31], ^e^Jeya et al. 2020 [52]

### Quantification of sphingolipids by liquid chromatography tandem-mass spectrometry (LC-MS/MS)

iBECs were seeded in 6-well plates on day 8 of differentiation at a density of 1 × 10^6^ cells per well. On day 10, when the cells reached confluency, they were either infected (MOI 100), treated with 10 mg/ml CPS, or left uninfected. After the designated infection period, the cells were harvested, resuspended in 0.5 ml methanol and subjected to sphingolipid extraction by addition of 1 ml methanol/chloroform (1:1, v: v) as described [53]. Supernatants were collected and mixed with 3 ml 1-butanol for lipid extraction as described [31]. For work-up of both sample types, the extraction solvent contained the internal standards d_7_-dihydrosphingosine (d_7_-dhSph), d_7_-sphingosine (d_7_-Sph), d_7_-sphingosine 1-phosphate (d_7_-S1P), 17:0 ceramide (Cer 17:0), d_31_-16:0 sphingomyelin (d_31_-SM 16:0), 17:0 glucosyl(β) ceramide (HexCer 17:0) and 17:0 lactosyl(β) ceramide (LacCer 17:0) (all from Avanti Polar Lipids, Alabaster, USA). Final extracts were subjected to LC-MS/MS sphingolipid quantification applying the multiple reaction monitoring (MRM) approach. Chromatographic separation was achieved on a 1290 Infinity II HPLC (Agilent Technologies, Waldbronn, Germany) equipped with a Poroshell 120 EC-C8 column (3.0 × 150 mm, 2.7 μm; Agilent Technologies) guarded by a pre-column (3.0 × 5 mm, 2.7 μm) of identical material. MS/MS analyses were carried out using a 6495 C triple-quadrupole mass spectrometer (Agilent Technologies) operating in the positive electrospray ionization mode (ESI+). Chromatographic conditions and settings of the ESI source and MS/MS detector have been published elsewhere [53]. The mass transitions used for analysis of sphingolipid subspecies are given in Supplementary Table S1. Peak areas of Cer, SM, HexCer and LacCer subspecies, as determined with MassHunter Quantitative Analysis software (version 10.1, Agilent Technologies), were normalized to those of their internal standards followed by quantification via external calibration. DhSph, Sph, and S1P were directly quantified via their deuterated internal standards. Data analysis was carried out as described in the legend for Fig. 6.

### Immunofluorescence staining of P-gp

On day 8 of differentiation, iBECs were seeded at a density of 2 × 10⁵ cells per well in 8-well µ-slides (ibidi, Cat. No. 80821). On the day of the experiment (day 10), the culture medium was replaced with EC– medium 1 h prior to infection. Cells were then infected with GFP-expressing *N. meningitidis* MC58 at a MOI of 100 for 4 h or left uninfected as controls.

Following infection, cells were washed once with PBS and fixed with 4% PFA for 15 min at room temperature without Triton X-100 permeabilization to preserve membrane integrity. Cells were then blocked in 10% fetal calf serum (FCS) in PBS for 1 h at room temperature and incubated overnight at 4 °C with a mouse monoclonal anti-P-glycoprotein antibody (clone F4, 1:50 dilution), which recognizes an extracellular epitope and thus labels surface-expressed P-gp [54].

The next day, cells were washed three times with PBS and incubated for 1 h at room temperature with Alexa Fluor 555–conjugated secondary antibody (1:200 dilution). To visualize the plasma membrane, cells were stained with wheat germ agglutinin (WGA) conjugated to Alexa Fluor Plus 405 (Invitrogen) at a concentration of 5 µg/mL in HBSS for 10 min. DAPI was used to counterstain nuclei. Slides were imaged using a Nikon Eclipse Ti2 confocal microscope under identical acquisition and exposure settings for all conditions. Image processing was performed in NIS Elements (version 5.02), using consistent channel settings and color adjustments across all samples, based on negative controls stained with secondary antibody only to confirm specificity and guide background correction.

To quantify surface P-gp signal, we calculated the Corrected Total Cell Fluorescence (CTCF) for the red (Alexa Fluor 555) channel using Fiji (ImageJ). After splitting the RGB image into individual color channels, the WGA (membrane) and DAPI channel (blue) was used to manually outline individual cells using the polygon selection tool. These regions of interest (ROIs) were saved and applied to the red channel (P-gp) to measure Integrated Density and Area for each cell. Background fluorescence was measured from a fluorescence-free region of the slide, and CTCF was calculated using the formula:

CTCF = Integrated Density − (Area × Mean background fluorescence).

All measurements were performed under identical acquisition and exposure settings to ensure comparability across samples.

### Statistics

GraphPad Prism 10.2.3 (GraphPad Software Inc.) was used for all statistical analysis. For pairwise comparison, a two-tailed unpaired Student’s t-test was applied. Comparisons involving more than two groups were conducted using one- or two-way ANOVA followed by Dunnett’s post hoc test to determine significance compared to control groups. Data are represented as mean ± standard deviation (SD). Statistical significance was accepted at a *p*-value < 0.05.

## Results

### *Neisseria meningitidis* impairs P-glycoprotein activity in brain endothelial cells

**Fig. 1:**
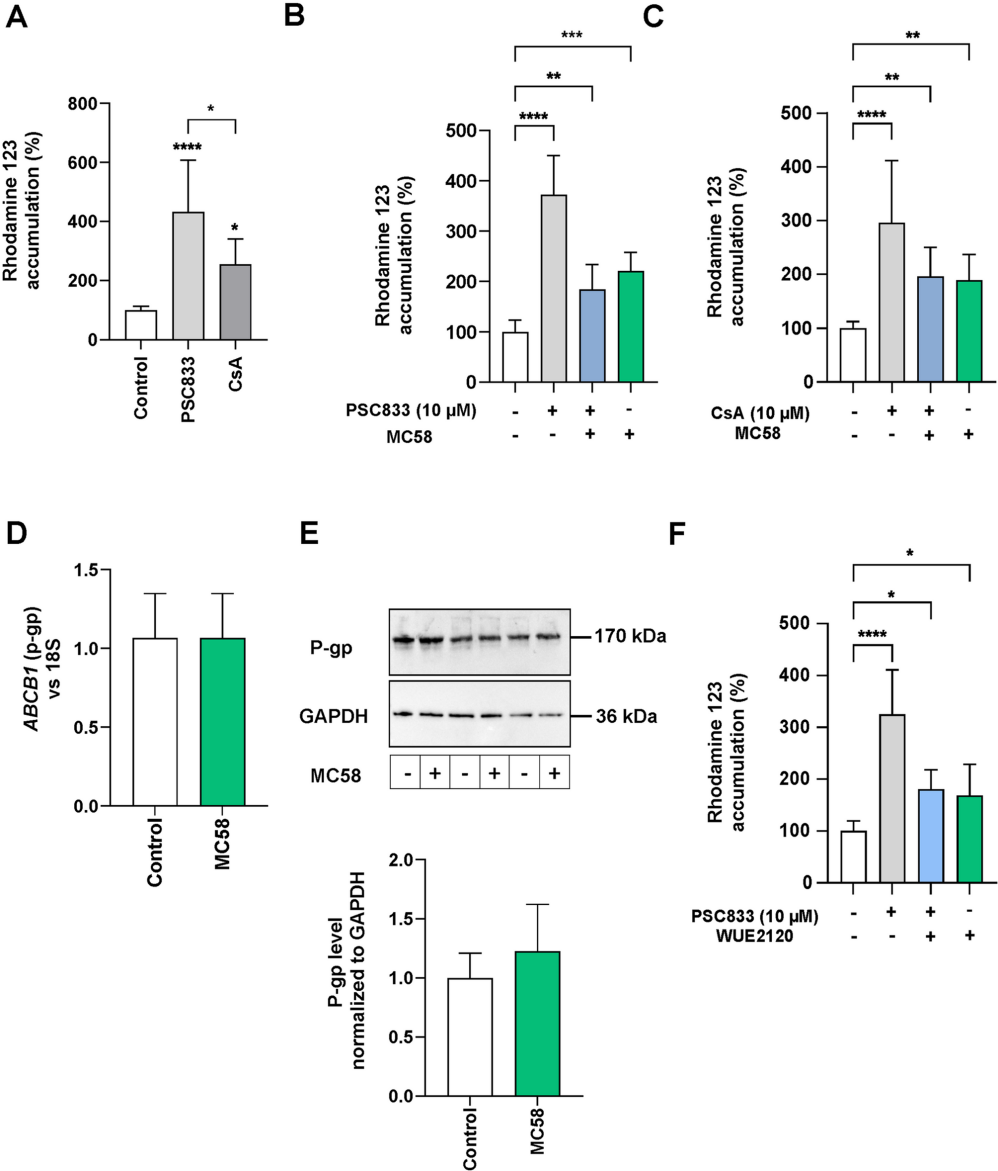
N. meninigitidis suppresses P-gp transport activity within iBECs without affecting mRNA or protein levels. (**A**) iBECs were pre-treated with specific P-gp inhibitors PSC833 (10 µM) or CsA (10 µM) for 1 h, followed by the addition of R123 (10 µM) for 2 h. Intracellular accumulation of R123 was measured to assess P-gp activity. (**B, C, F**) iBECs were either infected with *N. meningitidis* serogroup B strain MC58 (**B** and **C**) or serogroup C strain WUE2120 (**C**F) at an MOI of 100 for 4 h or left uninfected. P-gp activity was determined by measuring intracellular R123 accumulation. Results in panels A, B, C, and F are presented as the mean ± SD percentage change relative to the uninfected controls (white). PSC833- or CsA-treated cells served as positive controls. Statistical analysis for panel A was performed using Student’s t-test (PSC833 vs. CsA), while one-way ANOVA followed by Dunnett’s multiple comparison test was used for panels B, C, and F to compare all conditions to the uninfected controls. ****p < 0.0001, ***p < 0.001, **p < 0.01, *p < 0.05. (**D**) *ABCB1* (P-gp) expression was analyzed by RT-qPCR in iBECs infected with *N. meningitidis* MC58 (MOI 100) for 4 h. Expression levels were normalized to 18 S rRNA and data are presented relative to uninfected controls. (**E**) Total P-gp protein levels were determined using Western blot analyses. Densitometric analysis was performed using FIJI ImageJ software to quantify fold change in P-gp protein levels, which were normalized to GAPDH as loading control. Fold changes were calculated and expressed relative to uninfected controls. Data in panels D and E are presented as mean ± SD. Statistical differences were evaluated using Student’s t-test (infected vs. uninfected controls). All experiments shown in panels A-D and F were from three independent biological replicates conducted in triplicate, data shown in E were performed from three independent experiments

Brain endothelial cells (iBECs) were differentiated from induced pluripotent stem cells (iPSCs) and confirmed to express established markers characteristic of BECs as previously described (Fig. S1C) [36, 37, 55]. These iBECs were applied to investigate the impact of *N. meningitidis* on multidrug resistance (MDR) transporters, including P-glycoprotein (P-gp) and breast cancer resistance protein (BCRP). Treatment of iBECs with cyclosporin A (CsA) or Valspodar (PSC833), both selective inhibitors of P-glycoprotein (P-gp), resulted in a notable increase in intracellular Rhodamine 123 (R123) accumulation (Fig. 1A). This finding confirmed functional activity of P-gp in iBECs [16, 36]. In line with previous studies, PCS833, a more specific second-generation P-gp inhibitor, elicited a significant greater intracellular accumulation of R123 compared to CsA (Fig. 1A), demonstrating a greater efficacy in attenuating P-gp activity within our cell culture model [56, 57]. To determine the effect of *N. meningitidis* on P-gp activity, iBECs were infected with the wild-type (WT) *N. meningitidis* strain MC58 at an MOI of 100 for 4 h. This infection resulted in a significant increase in R123 accumulation (Fig. 1B, C), indicating a reduction in P-gp activity. In particular, infection with *N. meningitidis* resulted in approximately a twofold increase in R123 accumulation (Fig. 1B, C). Similar results were observed with *N. meningitidis* isolate WUE2120, a serogroup C meningococcal strain (Fig. 1F), suggesting that infection by meningococcal isolates from other serogroups similarly disrupts P-gp activity. Importantly, administration of PSC833 or CsA following infection did not further enhance R123 accumulation, suggesting that *N. meningitidis* infection alone interferes with P-gp inhibitor’s efficacy; however, the infection is insufficient to maximally inhibit P-gp activity (Fig. 1B, C).

To determine whether *N. meningitidis* infection had any effect on mRNA expression or P-gp protein levels, iBECs were infected with strain MC58 for 4 h as described before. Interestingly, unlike P-gp activity, both mRNA expression and protein levels of P-gp remained unchanged (Fig. 1D, E), indicating no direct correlation between P-gp activity and its protein abundance or gene expression.

### P-gp Inhibition is dependent on the level of bacterial interaction and viability

**Fig. 2:**
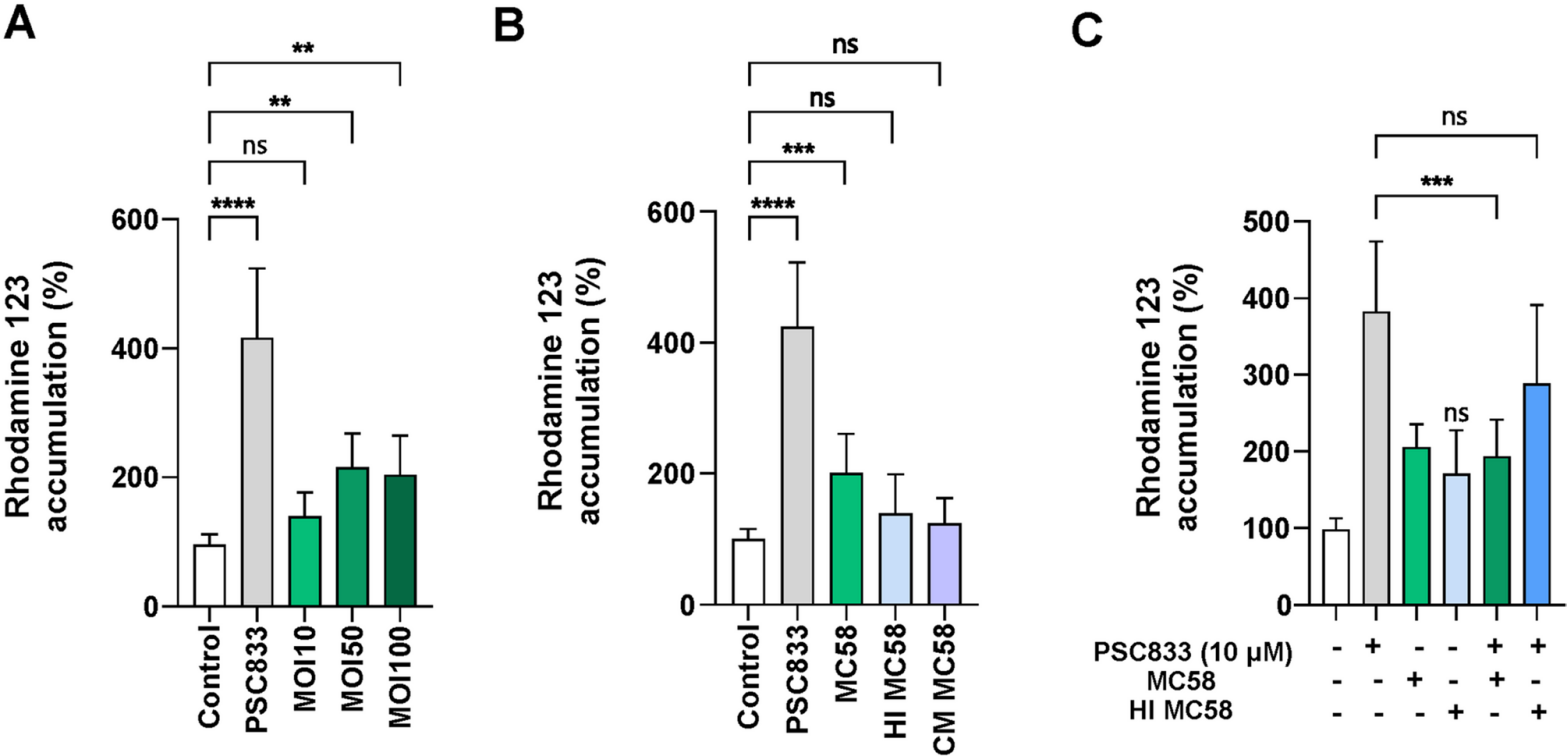
Bacterial interactions regulating P-gp inhibition in iBECs. (**A**) iBECs were infected with *N. meningitidis* strain MC58 at varying MOI (MOI 10, 50, 100) for 4 h, and Intracellular accumulation of R123 was measured. (**B**) The effects of live *N. meningitidis*, heat-inactivated (HI) *N. meningitidis*, and *N. meningitidis* MC58 conditioned-medium (CM) on R123 accumulation were evaluated after 4 h treatment. (**C**) iBECs were infected with live or HI *N. meningitidis* MC58 for 4 h, followed by treatment with or without PSC833, and P-gp functional activity was assessed relative to uninfected controls (white). Data are presented as a percentage of the uninfected controls (white), and PSC833-treated cells served as a positive control. All experiments were performed in triplicate with at least three independent biological replicates conducted in triplicate. Error bars indicate mean ± SD. Statistical significance was determined using one-way ANOVA followed by Dunnett’s multiple comparison test. ****p < 0.0001, ***p < 0.001, **p < 0.01, *p < 0.05, and ns (non-significant) indicating *p* > 0.05

We next investigated the impact of *N. meningitidis* on dysfunction of P-gp transporter activity at varying MOIs. Infection of the cells with *N. meningitidis* strain MC58 at an MOI of 10 for 4 h did not result in a significant increase in intracellular accumulation of R123 (Fig. 2A). In contrast, infections at MOIs of 50 and 100 substantially disrupted P-gp activity at 4 h p.i. (Fig. 2A). Additionally, infection at an MOI of 100 resulted in R123 accumulation levels comparable to those at an MOI of 50, suggesting that the inhibition of P-gp transporter activity by the bacteria reaches saturation at elevated infection levels.

To explore whether bacterial components or factors secreted by *N. meningitidis* contributed to P-gp inhibition, we next exposed iBECs to heat-inactivated (HI) *N. meningitidis* strain MC58 or to conditioned media (CM) derived from the same meningococcal isolate (Fig. 2B). The results showed that exposure to HI *N. meningitidis* MC58 did not significantly affect intracellular R123 accumulation, nor did treatment with *N. meningitidis*-CM alter P-gp activity compared to untreated controls (Fig. 2B, C). In addition, unlike live bacteria (Fig. 1B), HI bacteria did not interfere with PSC833 efficacy when added to the cells following the infection (Fig. 2C). These findings suggest that live bacterial interactions are required for the observed effect on P-gp activity.

**Fig. 3:**
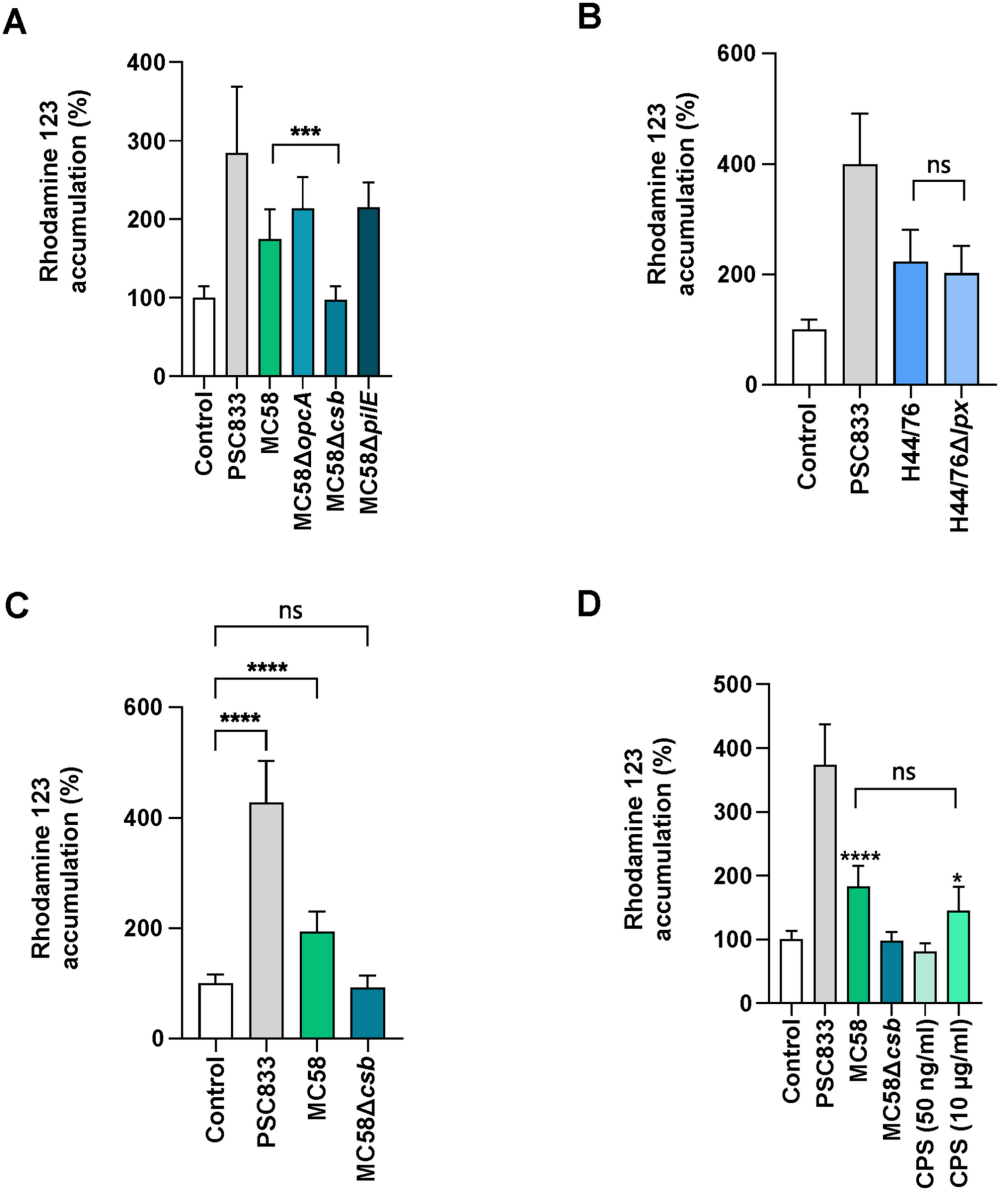
N. meningitidis capsular polysaccharide significantly contributes to inhibition of P-gp activity. (**A, B,** and **C**) iBECs were infected with WT or isogenic mutants (Δ*opcA*, Δ*csb*, Δ*pilE*, Δ*lpx*) of either *N. meningitidis* strain MC58 or strain H44/76. Infections were performed at an MOI of 100 for 4 h (**A, B**) or 6 h (**C**), and P-gp activity was determined by measuring intracellular R123 accumulation. (**D**) iBECs were infected with either WT strain MC58 or isogenic capsule-deficient mutant strain MC58Δ*csb* at an MOI of 100 or treated with purified CPS derived from *N. meningitidis* MC58 with indicated concentrations for 4 h. Experiments were performed at least in triplicate on three independent differentiations. Error bars represent mean ± SD. Statistical analysis between two groups was conducted using unpaired Student’s t-test (**D**) and for more than two groups one-way ANOVA followed by Dunnett’s multiple comparison test, comparing all conditions against uninfected controls (white). ****p < 0.0001, ***p < 0.001, *p < 0.05, ns *p* > 0.05

### *Neisseria meningitidis* capsular polysaccharide inhibits P-gp transporter activity

To assess the contribution of various virulence factors of *N. meningitidis* to the potential disruption of P-gp activity during infection, we next examined several isogenic mutants of strain MC58, including an isogenic capsule-deficient mutant (*N. meningitidis* MC58∆*csb*), an isogenic non-piliated mutant (*N. meningitidis* MC58∆*pilE*), and an isogenic mutant lacking outer membrane adhesion protein OpcA (*N. meningitidis* MC58∆*opcA*), each representing important virulence factors involved in the interaction of *N. meningitidis* with BECs during infection (Fig. 3A) [24, 30]. We also included *N. meningitidis* strain H44/76 and an isogenic lipooligosaccharide (LOS)-deficient mutant (*N. meningitidis* H44/76∆*lpx)* to determine a possible role of LOS (Fig. 3B), since LPS has been recently shown to modulate activity and expression of P-gp [13, 58, 59]. We observed that iBECs that were infected with either the non-piliated mutant MC58∆*pilE* or OpcA-deficient mutant MC58∆*opcA* showed similar levels of intracellular R123 accumulation compared to cells infected with WT strain MC58, suggesting that neither the pilus nor OpcA contribute to alteration of P-gp activity (Fig. 3A). In addition, LOS-deficient mutant *N. meningitidis* H44/76∆*lpx* exhibited increased intracellular accumulation of R123 to levels comparable to those observed with the WT strain (Fig. 3B). In contrast, the capsule-deficient mutant failed to increase intracellular R123 levels, demonstrating that it did not affect P-gp activity in BECs (Fig. 3A). To rule out that the effect was not due to differences in bacterial growth between the WT and the capsule-deficient mutant, bacterial growth kinetics of both the WT and the capsule-deficient mutant were assessed over a period of 6 h (Fig. S4C, D). We found the capsule mutant showed a slower growth rate, as indicated by lower colony forming units per ml (CFU/ml) in the cell culture medium after 4 h (Fig. S4C, D). By 6 h, as the WT strain entered the stationary growth phase, this difference in bacterial load was no longer statistically significant (Fig. S4C, D). To further assess the effect of the capsule, the experiments were repeated with a 6-hour infection period. The capsule-deficient mutant still failed to increase R123 levels within iBECs, whereas the WT strain consistently inhibited P-gp activity (Fig. 3C). These findings suggest that the capsule may contribute to the inhibition of P-gp activity. To further elucidate the role of the *N. meningitidis* CPS, CPS from *N. meningitidis* serogroup B strain MC58 was purified and applied to iBECs at varying concentrations. At a concentration of 10 µg/ml, purified CPS resulted in a twofold increase in R123 accumulation, mirroring the effect observed with the WT strain (Fig. 3D). Taken together, these results highlight the critical role of CPS in regulating P-gp activity in iBECs during *N. meningitidis* infection.

### Functional activity of BCRP remains unaffected during *N. meningitidis* infection

**Fig. 4:**
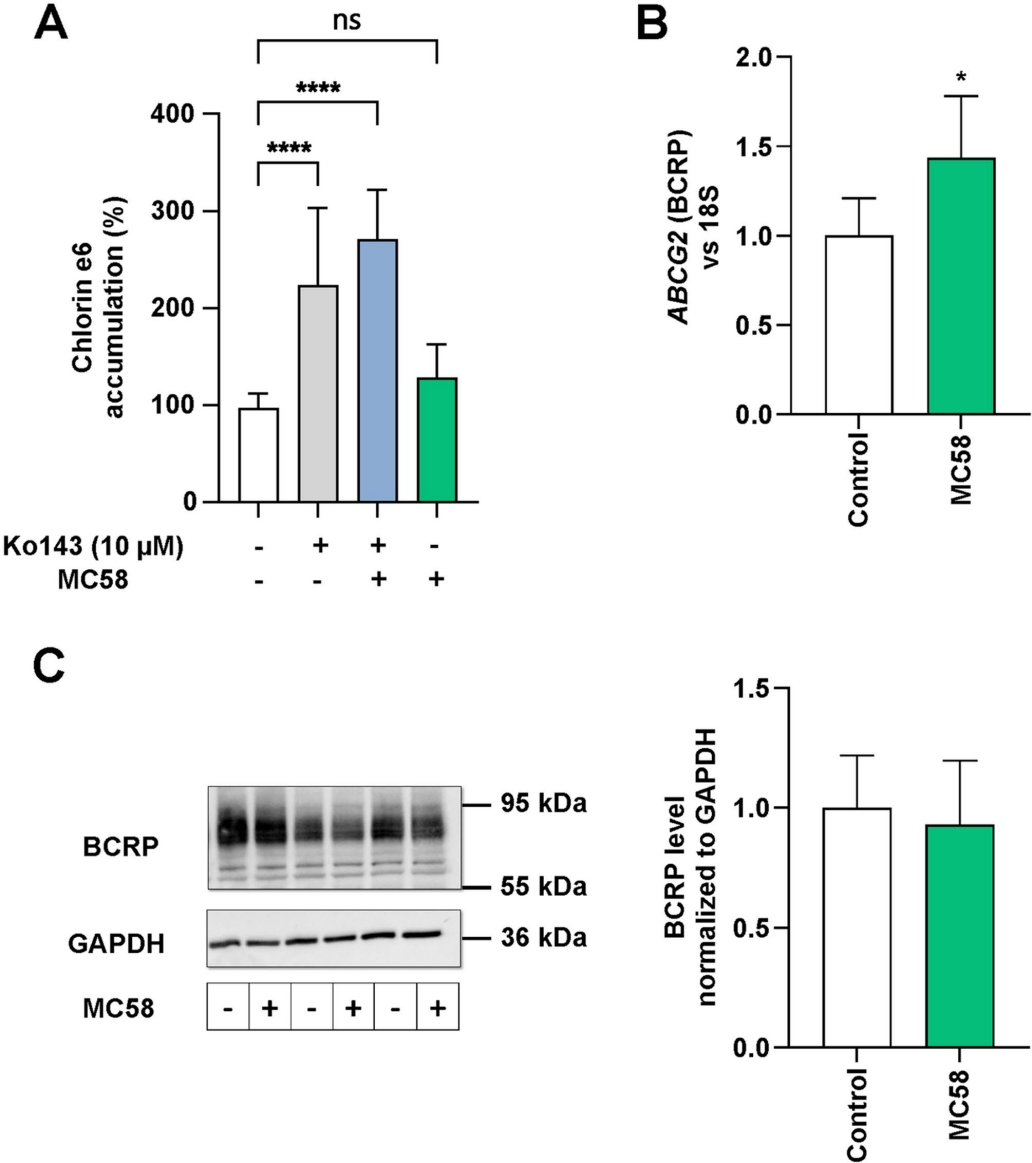
BCRP activity in iBECs remained unaffected 4 h after infection with *N. meningitidis*. (**A**) iBECs were either infected with *N. meningitidis* isolate MC58 at an MOI of 100 for 4 h or left uninfected. BCRP activity was determined by measuring intracellular Chlorin e6 accumulation. Ko143-treated cells served as positive controls. Data are expressed as a percentage of uninfected controls (white). Statistical analysis was conducted using one-way ANOVA followed by Dunnett’s multiple comparison test, comparing all conditions against the control. ****p < 0.0001, ns *p* > 0.05. (**B**) *ABCG2* (BCRP) mRNA levels were measured using RT-qPCR. Expression levels were normalized to 18 S rRNA and presented relative to uninfected controls (white). (**C**) Total BCRP protein levels were determined using Western blot analysis. Densitometric analysis was performed using FIJI ImageJ software to quantify total P-gp protein level changes which were normalized to GAPDH as loading control. Fold changes were calculated and expressed relative to the uninfected controls. Data in panels B and C are presented as mean ± SD. Statistical differences were evaluated using Student’s t-test (infected vs. uninfected controls), *p < 0.05. All experiments in A and B were conducted in triplicate with three independent biological replicates, experiments in C were from three independent biological replicates

In previous studies, infection mimics were found to have opposite effects on the functional activities of P-gp and the human BCRP in immortalized hCMEC/D3 cells, suggesting a potential compensatory relationship between these transporters in BECs [13]. Given our findings that *N. meningitidis* inhibited P-gp activity, we conducted a substrate accumulation assay to investigate the impact of *N. meningitidis* on BCRP in iBECs to explore a potential compensatory relationship between P-gp and BCRP that has already been suggested. Treatment of the cells with Ko143, a specific inhibitor for BCRP, resulted in a significant increase in intracellular accumulation of BCRP substrate chlorin e6 (1 µM), confirming the functional activity of this transporter in iBECs (Fig. 4A). iBECs were infected with strain *N. meningitidis* MC58 for 4 h as described, and accumulation of Ce6 was determined. No significant difference in Ce6 accumulation was observed between infected and uninfected cells at 4 h p.i. (Fig. 4A), indicating that BCRP activity remained unaffected at this time point. Additionally, infection of BECs with *N. meningitidis* did not exhibit the same interference with the BCRP inhibitor as observed with P-gp inhibitors (Fig. 4A). We also determined the effect of *N. meningitidis* infection on BCRP mRNA expression and protein levels. Our analysis demonstrated a slight, but significant increase in BCRP mRNA expression at 4 h p.i., whereas this elevation in BCRP mRNA expression did not correspond to changes in BCRP protein levels within infected iBECs (Fig. 4B, C).

### S1PR_1_ signaling reduces P-gp activity in *N. meningitidis* infected BECs

**Fig. 5:**
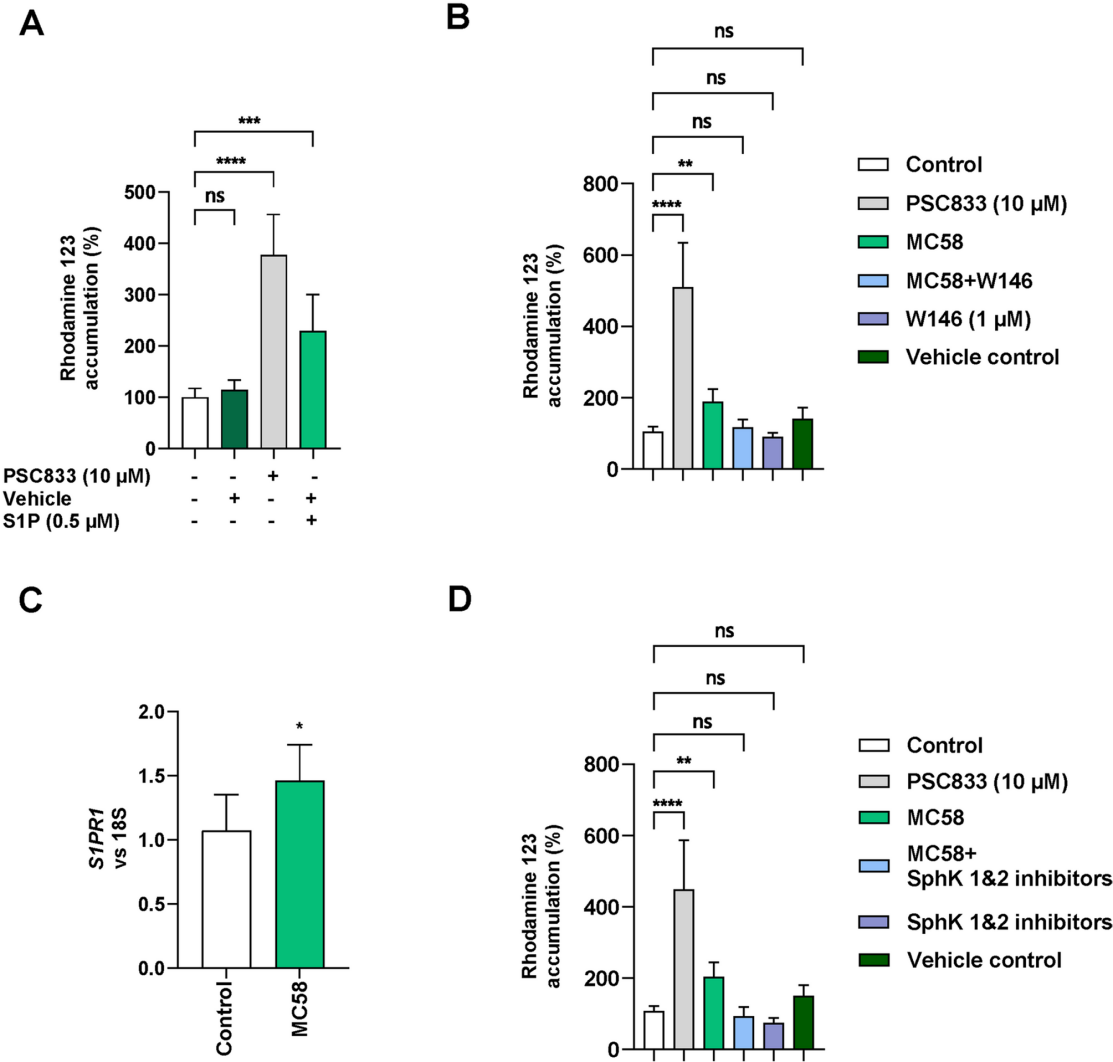
N. meningitidis infection reduces P-gp transport activity through S1P signaling via S1PR_1_. (**A**) The effect of S1P on P-gp activity was evaluated by measuring intracellular R123 accumulation. In the substrate accumulation assay, S1P (0.5 µM) or vehicle (4 mg/ml BSA in 1 × PBS) was added to the cells 20 min before analysis, coinciding with R123 incubation. (**B**) Blocking S1PR_1_ prevents *N. meningitidis*-induced reduction in P-gp activity. Cells were pre-treated with the S1PR_1_ antagonist W146 (1 µM) or vehicle control (DMSO) for 1 h, followed by infection with *N. meningitidis* (MOI 100) for 4 h, or cells remained uninfected. S1PR_1_ antagonist was maintained throughout the infection period. (**C**) Effect of *N. meningitidis* on mRNA expression of S1PR_1_. Relative mRNA expression level of *S1PR*_*1*_ was measured in the cells infected for 4 h. mRNA expression was analyzed using RT-qPCR, with normalization to 18 S rRNA and compared to uninfected controls. (**D**) Inhibition of sphingosine kinase (SphK) blocks the effects of *N. meningitidis* infection on P-gp activity. iBECs were pre-treated with SphK inhibitors (PF543 (10 µM) or SLM6031434 (1 µM)), or with vehicle control (DMSO), for 60 min. Cells were subsequently infected with *N. meningitidis* (MOI 100) for 4 h or remained uninfected. SphK inhibitors were maintained throughout the infection period. P-gp activity was determined by measuring intracellular R123 accumulation. All experiments were performed at least in triplicate on three independent differentiations. Data are presented as mean ± SD. In panels A, B and D, data are expressed as a percentage of the uninfected controls (white). Statistical analysis was conducted using one-way ANOVA followed by Dunnett’s multiple comparison test, comparing all conditions against the uninfected controls. ****p < 0.0001, ***p < 0.001, **p < 0.01, *p < 0.05, ns *p* > 0.05. Data in C represent the mean ± SD of three independent biological replicates, with each replicate measured in triplicate and analyzed in duplicate. Statistical significance was determined using an unpaired Student’s t-test, *p < 0.05

**Fig. 6:**
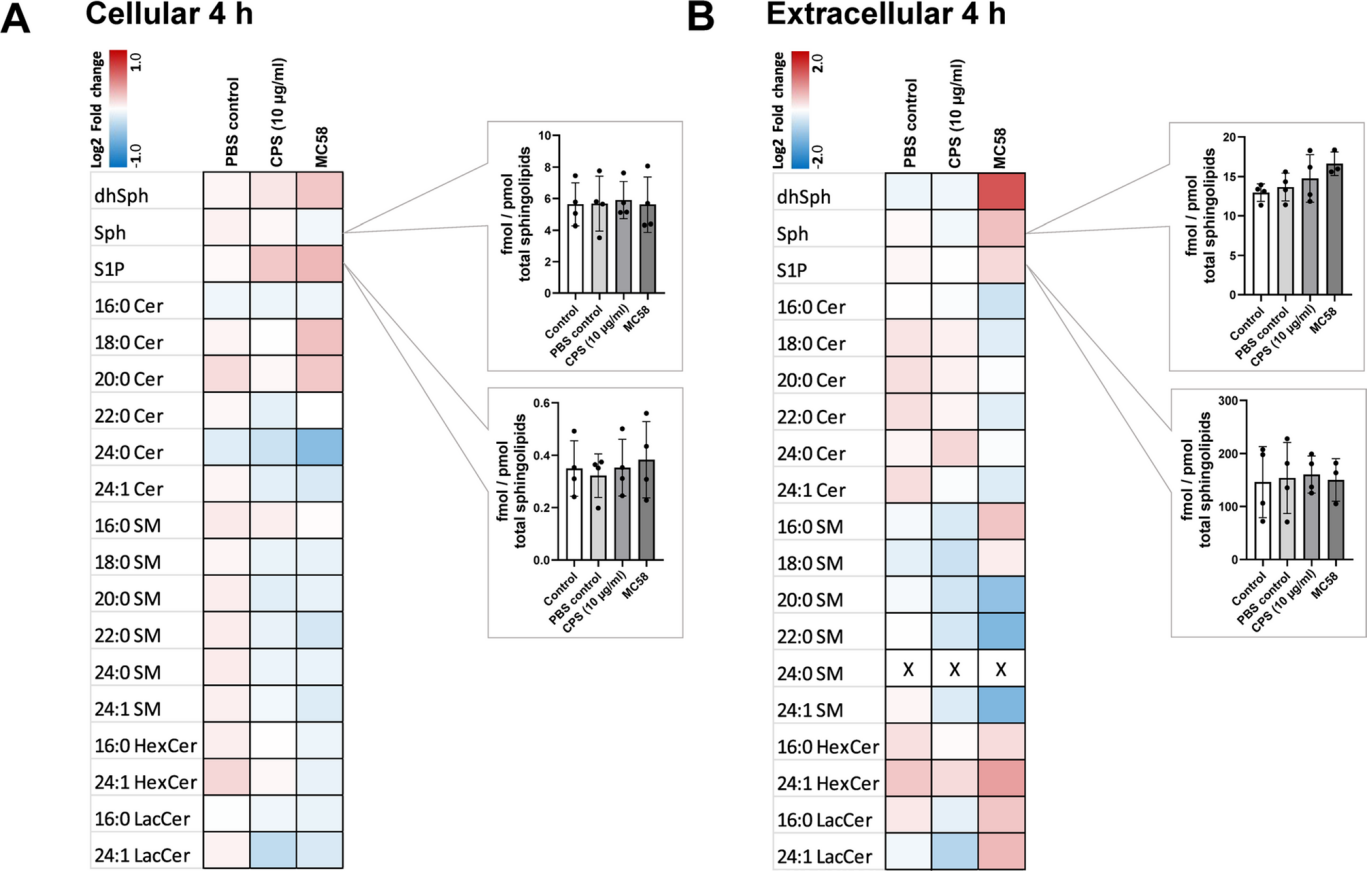
P-gp inhibition by *N. meningitidis* does not correlate with detectable changes in S1P. iBECs were infected with *N. meningitidis* MC58 at an MOI of 100, treated with CPS from the same isolate (10 µg/ml), or treated with 0.03 x PBS in EC– for 4 h. Sphingolipid metabolite levels were quantified at the indicated time points by LC-MS/MS for both cellular (**A**) and extracellular (**B**) samples. Heatmaps show the log2 fold-change values of measured sphingolipid metabolites relative to uninfected controls from at least three independent biological replicates, and log2 fold-change scales are displayed in the corner of each heatmap. The graphs indicate sphingolipid metabolite levels relative to total sphingolipids (fmol/pmol total sphingolipid). Data are presented as mean ± SD from at least three independent biological replicates. Statistical analysis was performed using one-way ANOVA followed by Dunnett’s multiple comparison test, comparing all conditions against the uninfected controls (white)

Our data indicated that P-gp is likely regulated through an indirect mechanism that influences its activity without affecting its expression levels. The identified regulatory mechanisms of P-gp transporter activity encompass sphingolipid signaling, vascular endothelial growth factor (VEGF) signaling leading to removal of P-gp from the membrane or ubiquitin-activating enzyme E1 degradation and ubiquitination [60]. Research conducted by Miller and colleagues observed that sphingosine 1-phosphate (S1P) via S1P receptor 1 (S1PR_1_) reversibly reduced P-gp activity in rat and mice brain capillaries [21, 22]. Interestingly, we recently demonstrated that *N. meningitidis* enhanced extracellular levels of S1P levels in hCMEC/D3 cells [31]. Based on these findings, we hypothesized that P-gp activity is regulated via S1P and S1PR_1_ signaling in iBECs in response to *N. meningitidis* infection. We first tested whether S1P treatment of iBECs could modulate P-gp activity and found that, consistent with published studies, 0.5 µM S1P treatment for 20 min significantly increased R123 levels, thus demonstrating decreased P-gp function (Fig. 5A). S1P exerts its effects through five extracellular receptors (S1PR_1–5_), with S1PR_1_ signaling specifically implicated in the regulation of P-gp activity [21, 22, 61, 62]. To investigate the functional role of S1PR_1_ in reduced P-gp transport activity in iBECs during *N. meningitidis* infection, cells were treated with the S1PR_1_ antagonist W146 (1 µM) (Fig. 5B). Treatment of iBECs with W146, prevented the infection-induced accumulation of R123, indicating a reversal of the inhibition of P-gp activity (Fig. 5B). Note, that W146 (at 1 µM) by itself caused small but not significant reduction of intracellular accumulation of R123, indicating slight S1PR_1_-mediated P-gp inhibition under control conditions. Using sequence-targeted primers of each of the human S1PR_1 − 3_ transcripts in a RT-PCR assay we observed significant upregulation of S1PR_1_ in iBECs infected with *N. meningitidis* (MOI 100, 4 h), while S1PR_2_ expression remained unaffected and S1PR_3_ expression significantly decreased at 4 h p.i. (Figs. 5C, S6). Infection of hCMEC/D3 cells with *N. meningitidis* has been shown to stimulate S1P production from sphingosine through activation of sphingosine kinases (SphK) [31, 63, 64]. We infected iBECs with *N. meningitidis* in the absence and presence of SphK1 and Sphk2 inhibitors (PF543, 10 µM, and SLM6031434, 1 µM, respectively). Treatment with both SphK inhibitors, blocked *N. meningitidis*-induced R123 accumulation (Fig. 5D) and thus the reduction in transporter activity. Of note, SphK inhibitors themselves caused small, but not significant reductions in intracellular accumulation of R123 (Fig. 5D). Next, targeted quantitative measurements of sphingolipids were performed at 4 h p.i. in iBECs, either left uninfected (control) or infected with *N*. *meningitidis* strain MC58 or treatment with purified CPS, and in their supernatants to determine whether the effects are associated with increased levels of S1P and/or sphingosine. Data analysis revealed no significant effect on cellular as well as extracellular levels of both sphingosine (Sph) or S1P following 4 h of infection with *N. meningitidis* MC58 or treatment with purified CPS (Fig. 6A, B, Heatmaps). Analysis of metabolite levels relative to the total sphingolipid profile also demonstrated no significant changes in Sph or S1P levels under any of these conditions (Fig. 6A, B). In contrast, extracellular dihydrosphingosine (dhSph) levels increased by 1.3-fold following 4 h of infection with strain MC58. However, this increase was not statistically significant when normalized to total sphingolipid levels (data not shown).

## Discussion

An essential characteristic of ECs at the mBSCFB or BBB is the formation of interendothelial TJ complexes, which contribute to high TEER and are associated with the expression of key proteins such as occludin and claudin-5 [1]. Additionally, BECs express several membrane transporters, including MDR transporters such as P-gp, which serve as critical components of the BBB by restricting the entry of many drugs and neurotoxic compounds. While *N. meningitidis* infection has been shown to disrupt or reorganize TJ proteins [34, 35, 49, 65], thereby compromising endothelial barrier integrity at later stages of infection, the impact of this pathogen on MDR transporters has not been explored.

The present study demonstrates that *N. meningitidis* infection impairs the transport activity of the MDR transporter P-gp in BECs derived from induced pluripotent stem cells (iBECs), without altering P-gp mRNA expression or protein levels. We also show that the effect on P-gp activity is dependent on the bacterial viability and CPS and is mediated through sphingolipid signaling pathways involving S1PR_1_. The decrease in P-gp activity observed in this study occurred without any detectable changes in P-gp expression or protein abundance at 4 h p.i. This finding is in line with previous studies that have reported a disconnect between P-gp activity and its expression level or protein abundance, suggesting that the regulation of P-gp activity can be independent of its overall protein levels in the cell [13, 66]. P-gp can be modulated through post-translational modifications, such as phosphorylation, glycosylation, or ubiquitination, which influence the conformation of the transporter, and ultimately lead to changes in its functional activity [21, 60, 67–72]. Here, we demonstrate that *N. meningitidis* likely modulates P-gp functional activity through sphingolipid signaling via S1PR_1_.

S1P, a bioactive phospholipid signaling molecule, is abundantly present in plasma and plays a pivotal role in regulating the barrier function of BECs [31, 73]. Alterations in sphingolipid levels, particularly S1P, have been previously documented during *N. meningitidis* infection in the immortalized cell line hCMEC/D3 [31]. Our findings here demonstrate that S1PR_1_ signaling constitutes a critical regulator in the inhibition of P-gp activity during *N. meningitidis* infection of iBECs. Treatment of iBECs with S1P resulted in a significant increase in R123 accumulation, indicating reduced P-gp activity in response to sphingolipid signaling as previously reported by Miller and colleagues [21, 22, 62]. Blocking S1PR_1_ with the specific antagonist W146 impaired the inhibitory effect of *N. meningitidis* on P-gp activity supporting the importance of S1PR_1_ in this process. Pharmacological inhibition of SphK prevented the infection-induced reduction in P-gp activity, and notably, SphK inhibition in uninfected cells decreased R123 accumulation by about 30%, suggesting increased P-gp activity when S1P synthesis is blocked in BECs. To further investigate this pathway, we used LC-MS/MS to quantify the S1PR_1_ ligand S1P in the supernatant of infected iBECs, but failed to detect significant release of S1P. One possible explanation for the lack of any increase in S1P levels in both the cell lysate and supernatant following infection or treatment with CPS compared to controls might be that the LC-MS/MS analysis was conducted at an incorrect time point, potentially missing a transient peak in S1P levels. S1P may be released within a short time window after infection or treatment, after which it is rapidly metabolized. To address this, a time-course experiment assessing S1P kinetics would identify optimal sampling time points, providing more precise temporal data and potentially revealing transient changes in S1P concentration that were not captured in the current experimental design. Otherwise, a spatially restricted release of S1P may be sufficient to signal through an autocrine mechanism via S1PR_1_ without leading to detectable concentration differences in the supernatant sampled for analysis. Of note, an increase in the expression of S1PR_1_ was observed in *N. meningitidis*-infected iBECs. This finding suggests that signaling through S1PR_1_ could occur even in the absence of a measurable increase in S1P levels. The increased receptor expression may indicate activation of signaling pathways that are independent of S1P concentration, possibly through altered receptor density or enhanced signaling from S1PR_1_ itself.

Our findings demonstrated that *N. meningitidis* selectively inhibited P-gp activity in BECs, while the activity of BCRP remained unaffected. This selective modulation may arise from structural and functional differences between the two transporters: P-gp is a full transporter consisting of two membrane-spanning domains (MSDs) and two intracellular ATP-bindings regions (nucleotide-binding domains; NBDs). In contrast, BCRP is a half-transporter, containing only one MSD and one NBD [74–76]. BCRP is known to form homodimers or higher-order oligomers, and it is suggested to have multiple binding sites [75, 77]. Disruption of interactions between BCRP monomers has been proposed to impede the formation of active oligomers, thereby inhibiting its activity [78]. However, the redundancy provided by its multiple binding sites may enable BCRP to maintain functionality, even in the presence of pathogen-induced stressors. The differential modulation of these transporters may also reflect variations in their regulatory mechanisms. P-gp appears to be highly sensitive to S1PR_1_-mediated signaling, which has been shown to reduce its efflux activity, potentially by altering its localization or functional state. In contrast, BCRP does not appear to be influenced by S1P/S1PR_1_ signaling, suggesting that its oligomeric structure and substrate-binding redundancy confer greater stability under infection-induced changes. These findings highlight distinct regulatory pathways governing P-gp and BCRP activity and provide insight into the selective effects of *N. meningitidis* on efflux transporters at the mBCSFB.

Our results showed that the bacterial capsule is essential for the inhibition of P-gp activity by *N. meningitidis*. Deletion of the *csb* gene, which encodes the polysialyltransferase responsible for synthesizing the α (2→8)-linked sialic acid homopolymer of the serogroup B CPS, completely abolished P-gp inhibition. Importantly, purified CPS from serogroup B strain MC58 inhibited P-gp activity in iBECs in a dose-dependent manner, with significant effects observed at 10 µg/ml. This finding highlights the crucial role of CPS in this process. Exposure to HI bacteria still resulted in a slight increase in R123 accumulation (40–70%). In our hands, CPS was detectable in HI bacterial samples using ELISA (Fig. S2B), furthermore, underlining that CPS is necessary to achieve effective P-gp inhibition during infection. By using an additional strain, meningococcal serogroup C isolate WUE2120, we showed that this strain also caused a significant reduction in P-gp activity. CPS of *N. meningitidis* serogroup C is composed of homopolymers of sialic acid, with minor differences in structural linkages (α(2→9)-linked sialic acid homopolymers) and the partial O-acetylation of sialic acid [79]. These findings strongly support our observations that sialic-acid-based CPS of *N. meningitidis* triggers cellular signaling pathways that lead to modification of P-gp, inhibiting its activity.

However, the precise mechanisms underlying S1PR_1_ activation, and the downstream pathways mediating P-gp inhibition in BECs are not fully understood. Previous studies from our group have identified that *N. meningitidis* components, including the type IV pilus and OpcA protein, trigger activation of different enzymes of the sphingolipid metabolic pathways (SphK and acid sphingomyelinase, respectively) in either the immortalized cell lines HBMEC or hCMEC/D3 [31, 63, 80]. In the current study, using iBECs CPS emerged as a key factor in the inhibition of P-gp activity. Our findings suggest that CPS may facilitate the activation of S1PR_1_ to trigger downstream pathways resulting in altered P-gp activity, however, we cannot conclude from the data presented here that CPS enhances SphK activity.

Work from the laboratory of David Miller has defined a signaling pathway in rat brain capillaries that rapidly and reversibly reduces basal P-glycoprotein transport activity without changing transporter protein expression. In this pathway, TNF-*α* signals through tumor necrosis factor receptor 1, endothelin receptor B, inducible nitric-oxide synthase, and protein kinase C (PKCβ1) resulting in increased SphK activity [21, 62]. It is likely that CPS from *N. meningitidis* activate this signaling cascade, as *N. meningitidis* has been shown to induce TNF-α production in endothelial cells upon close contact with the host cell [81]. TNF-α, a central mediator of inflammation during *N. meningitidis* infections, plays a pivotal role in vascular dysfunction and immune activation. Supporting this, prior studies in HBMEC demonstrated significantly elevated TNF-α concentrations in supernatants of cells infected with the WT MC58 strain compared to an unencapsulated mutant [81].

S1PR_1_ is a G-protein-coupled receptor capable of activating several distinct signaling pathways, including PI3K/protein kinase B (Akt), phospholipase C/PKC, Ras/ERK, and adenyl cyclase/cAMP [22]. Further investigation is required to determine which of these pathways modulates P-gp activity in BECs in response to *N. meningitidis* infection. Apart from signal transduction two primary mechanisms have been proposed to account for reduced plasma membrane transporter activity: transporter internalization and alterations in the plasma membrane microenvironment [22]. In line with this, our immunofluorescence analysis (Fig. S7) revealed a trend toward reduced surface-localized P-gp in infected iBECs, suggesting possible redistribution of the transporter away from the membrane during *N. meningitidis* infection. While not statistically significant, this observation supports the hypothesis that changes in host membrane composition by *N. meningitidis* may influence P-gp trafficking and activity in BECs.

We recently established iBECs, derived from induced pluripotent stem cells, as a novel cellular model to investigate the interaction between *N. meningitidis* and BECs [49, 65]. This model provides a physiologically relevant representation of human BECs, offering a closer resemblance to primary BECs compared to commonly used immortalized cell lines [49]. The distinction is particularly significant because variations in cellular signaling pathways or receptor expression among different models can profoundly influence the dynamic interplay between CPS, type IV pili or OpcA and sphingolipid signaling. Unlike primary BECs, hCMEC/D3 cells exhibit markedly lower expression levels of P-gp [82]. This reduced expression may affect key physiological processes, including barrier integrity, drug efflux, and interactions with bacterial pathogens. By contrast, iBECs bridge this gap, offering a model that is both scalable and more representative of primary human BECs, thereby enabling more accurate investigations into the molecular mechanisms governing *N. meningitidis-*host cell interactions.

At present, we do not fully understand the significance of the reduction of P-gp activity in BECs in response to *N. meningitidis* infection. P-gp is primarily located at the luminal surface of BECs, where it plays a crucial role in restricting or preventing the brain entry of a variety of small lipophilic components, including drugs, toxins, and other harmful substances. Its efflux function acts as a protective barrier, maintaining the integrity of the mBCSFB ensuring that potentially harmful compounds do not accumulate in the brain. Thus, the abrogation of proper P-gp activity during *N. meningitidis* infection could potentially facilitate the entry of neurotoxic substances, further contributing to the pathogenesis of meningococcal meningitis.

## Conclusion

In this study, we identified *N. meningitidis* CPS as a key factor in impairing P-gp activity in BECs through the S1PR_1_ signaling pathway. This inhibition occurred independently of changes in P-gp expression or protein abundance and was specific to P-gp, as BCRP function remained unchanged. While our data clearly showed that CPS mediated this effect, the precise mechanisms—such as potential contribution from sphingosine kinase activity or other bacterial components like type IV pili—remain to be further investigated. By employing iBECs, a physiologically relevant model of the mBCSFB, our study provides novel insights into the molecular mechanisms underlying *N. meningitidis-*induced mBCSFB dysfunction. These findings suggest that targeting sphingolipid signaling pathways could represent a promising therapeutic strategy to restore P-gp function and preserve barrier integrity in neuroinflammatory conditions like meningococcal meningitis.

## Electronic supplementary material

Below is the link to the electronic supplementary material.

Supplementary Material 1

Supplementary Material 2

Supplementary Material 3

Supplementary Material 4

Supplementary Material 5

Supplementary Material 6

Supplementary Material 7

Supplementary Material 8

## Data Availability

The datasets used and/or analyzed during the current study are available from the corresponding author on reasonable request.

## Abbreviations

BBB: Blood-brain barrier
BCRP: Breast cancer resistance protein
BECs: Brain endothelial cells
bFGF: Basic fibroblast growth factor
BSA: Bovine serum albumin
CA: Colominic acid
Ce6: Chlorin e6
CFU/ml: Colony-forming unit per milliliter
CNS: Central nervous system
CPS: Capsular polysaccharide
CsA: Cyclosporin A
DAPI: 4′,6-diamidino-2-phenylindole
DMSO: Dimethyl sulfoxide
EC–: Endothelial cell assay medium
ECL: Enhanced chemiluminescence
ELISA: Enzyme-linked immunosorbent assay
EtOH: Ethanol
FCS: Fetal calf serum
hESFM: Human endothelial serum-free medium
HI: Heat-inactivated
iBECs: iPSC-derived brain-like endothelial cells
iPSC: Induced pluripotent stem cell
kDa: Kilodalton
LC-MS/MS: Liquid chromatography tandem-mass spectrometry
LDH: Lactate dehydrogenase
LOS: Lipopolysaccharide
mBCSFB: Meningeal blood-cerebrospinal fluid barrier
MDR: Multidrug resistance
MOI: Multiplicity of infection
OD: Optical density
P-gp: P-glycoprotein
PBS: Phosphate-buffered saline
PFA: paraformaldehyde
p.i.: post-infection
PPM: Proteose peptone medium
R123: Rhodamine123
RA: Retinoic acid
RIPA: Radioimmunoprecipitation assay buffer
RNA: Ribonucleic acid
RT: Room temperature
S1P: Sphingosine 1-phosphate
SDS: Sodium Dodecyl Sulfate
SphK: Sphingosine kinase
TBS-T: Tris-buffered saline with Tween 20
TEER: Transendothelial electrical resistance
TJs: Tight junctions
TX-100: Triton X-100
UM: Unconditioned medium
vs: Versus
WT: Wild-type
ZO-1: Zonula occludens-1
