# Supplementary figures and images for: Neisseria meningitidis regulates P-glycoprotein transporter activity in brain endothelial cells via sphingosine 1–phosphate receptor 1
Source: Fluids Barriers CNS. 2025 Jul 22;22:78. doi: 10.1186/s12987-025-00687-0 (PMC12285036; doi:10.1186/s12987-025-00687-0)

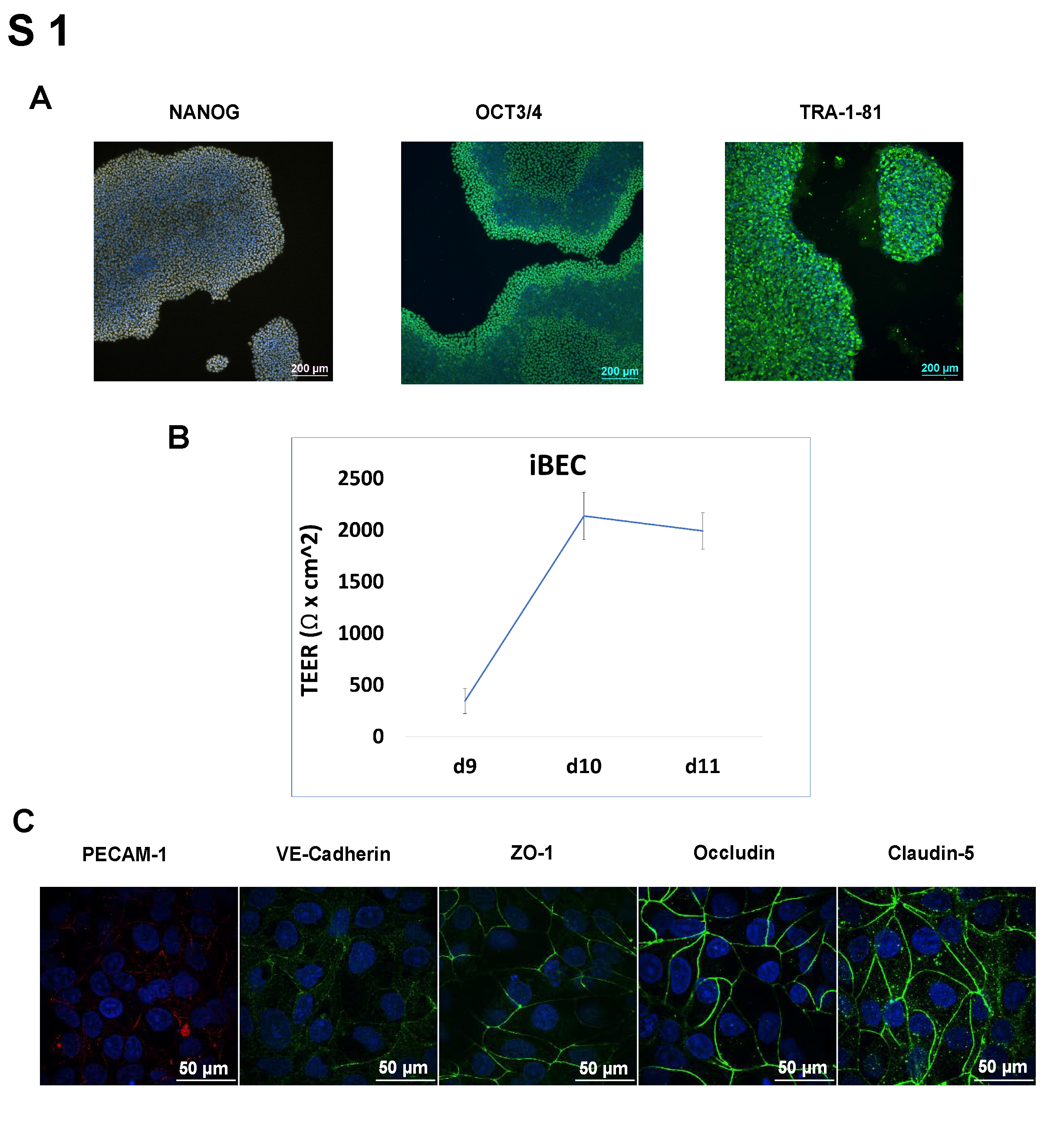

Supplement: Supplementary file 1 — Supplementary Material 1 [file 12987_2025_687_MOESM1_ESM.tif]

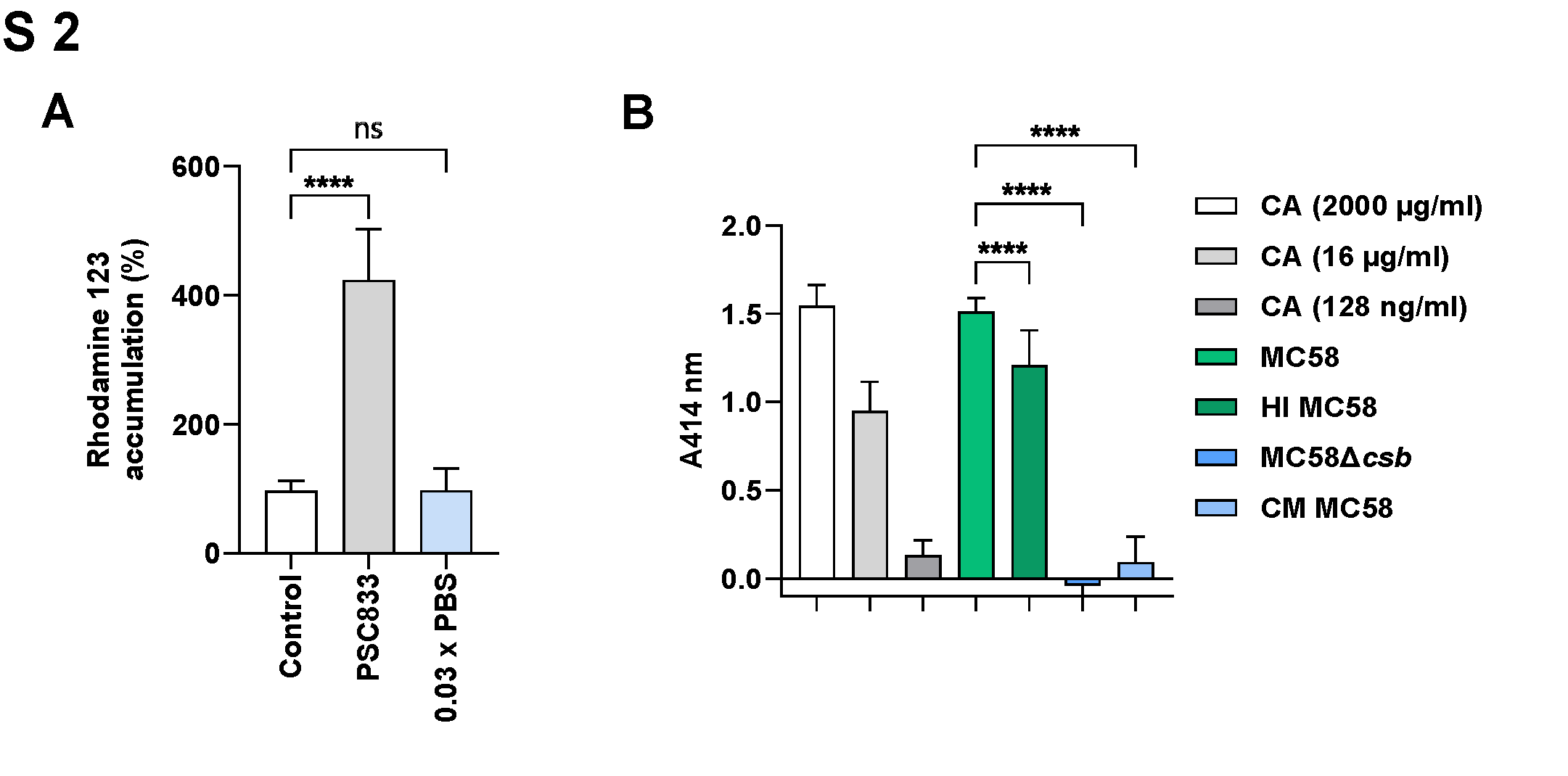

Supplement: Supplementary file 2 — Supplementary Material 2 [file 12987_2025_687_MOESM2_ESM.tif]

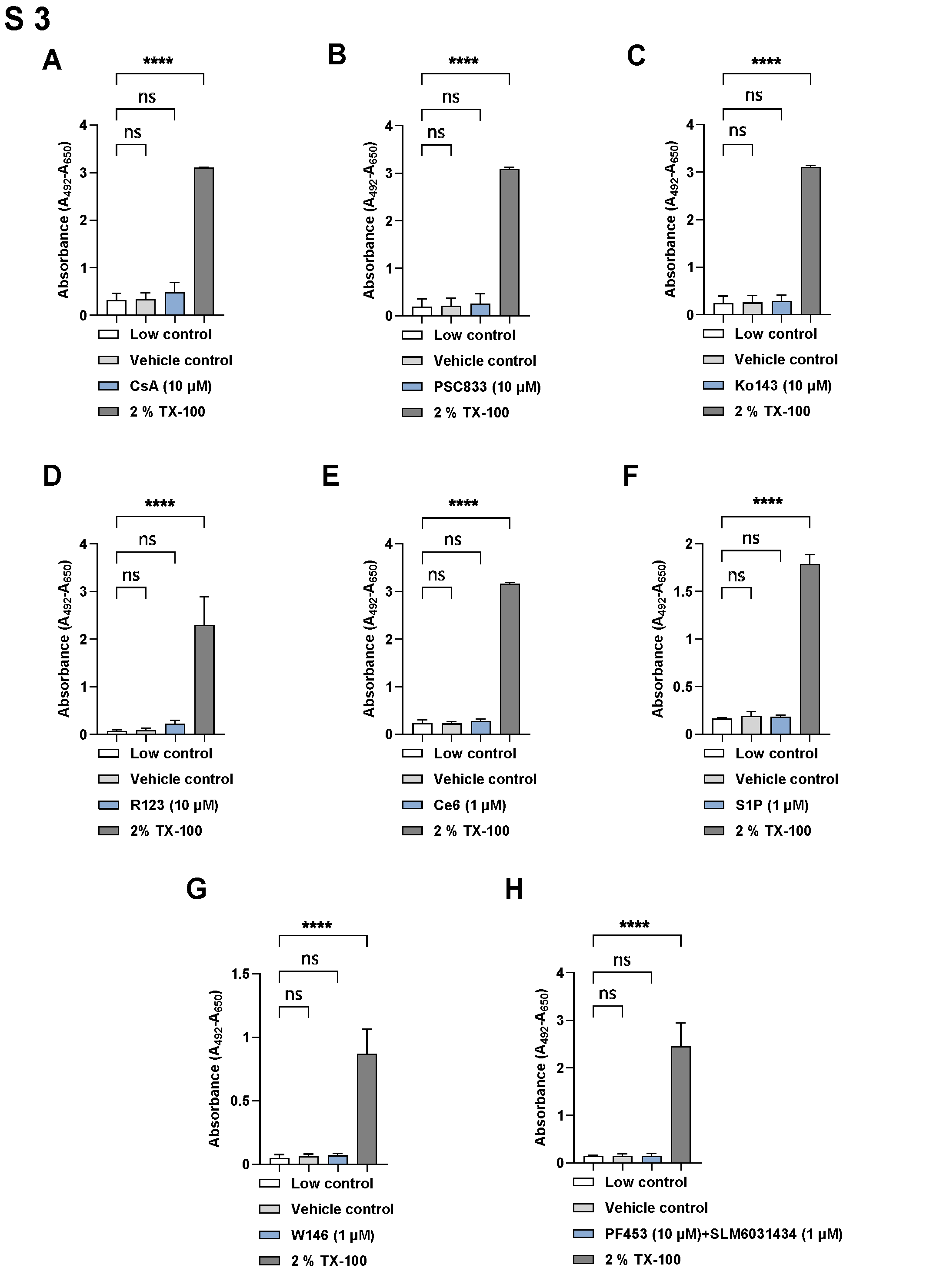

Supplement: Supplementary file 3 — Supplementary Material 3 [file 12987_2025_687_MOESM3_ESM.tif]

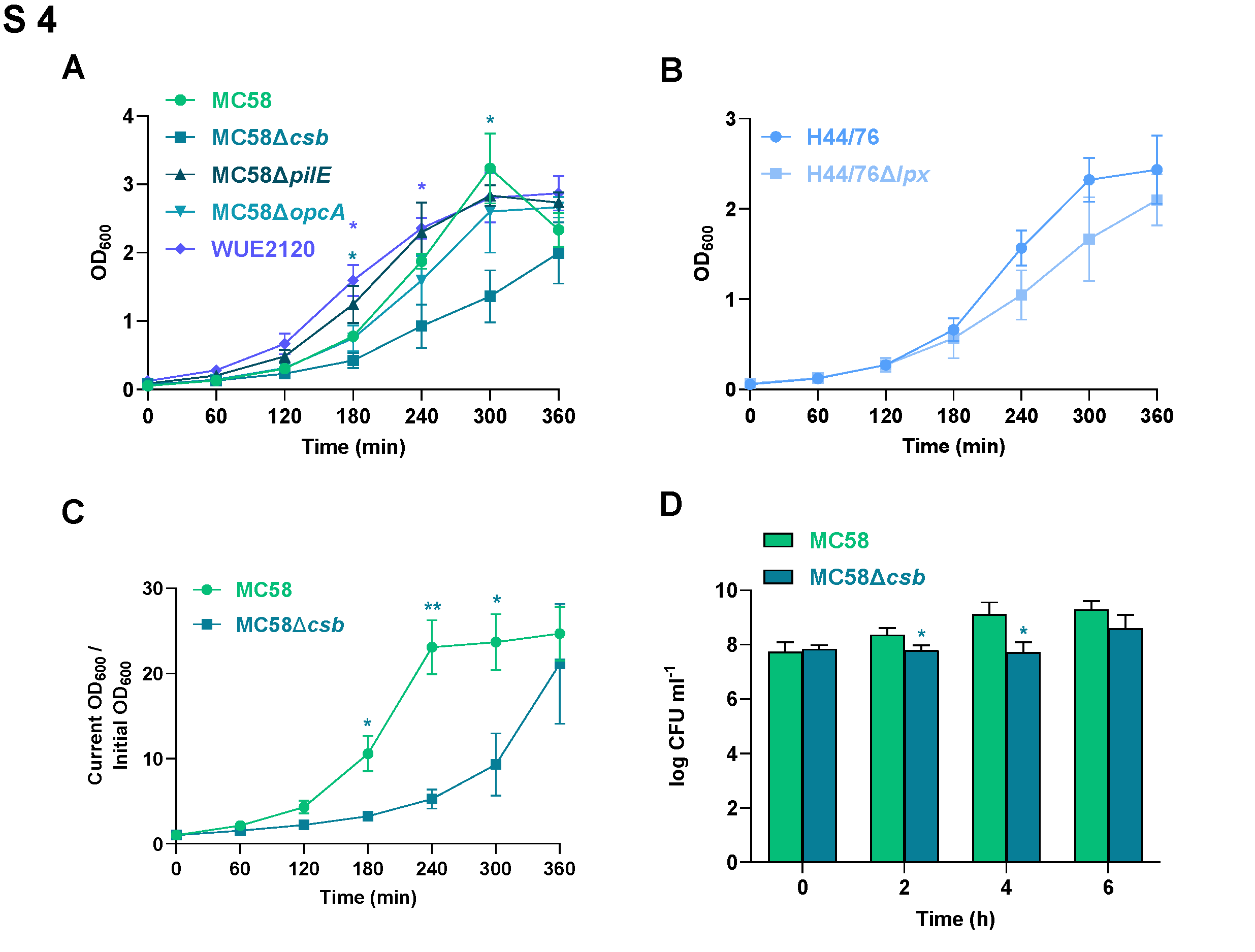

Supplement: Supplementary file 4 — Supplementary Material 4 [file 12987_2025_687_MOESM4_ESM.tif]

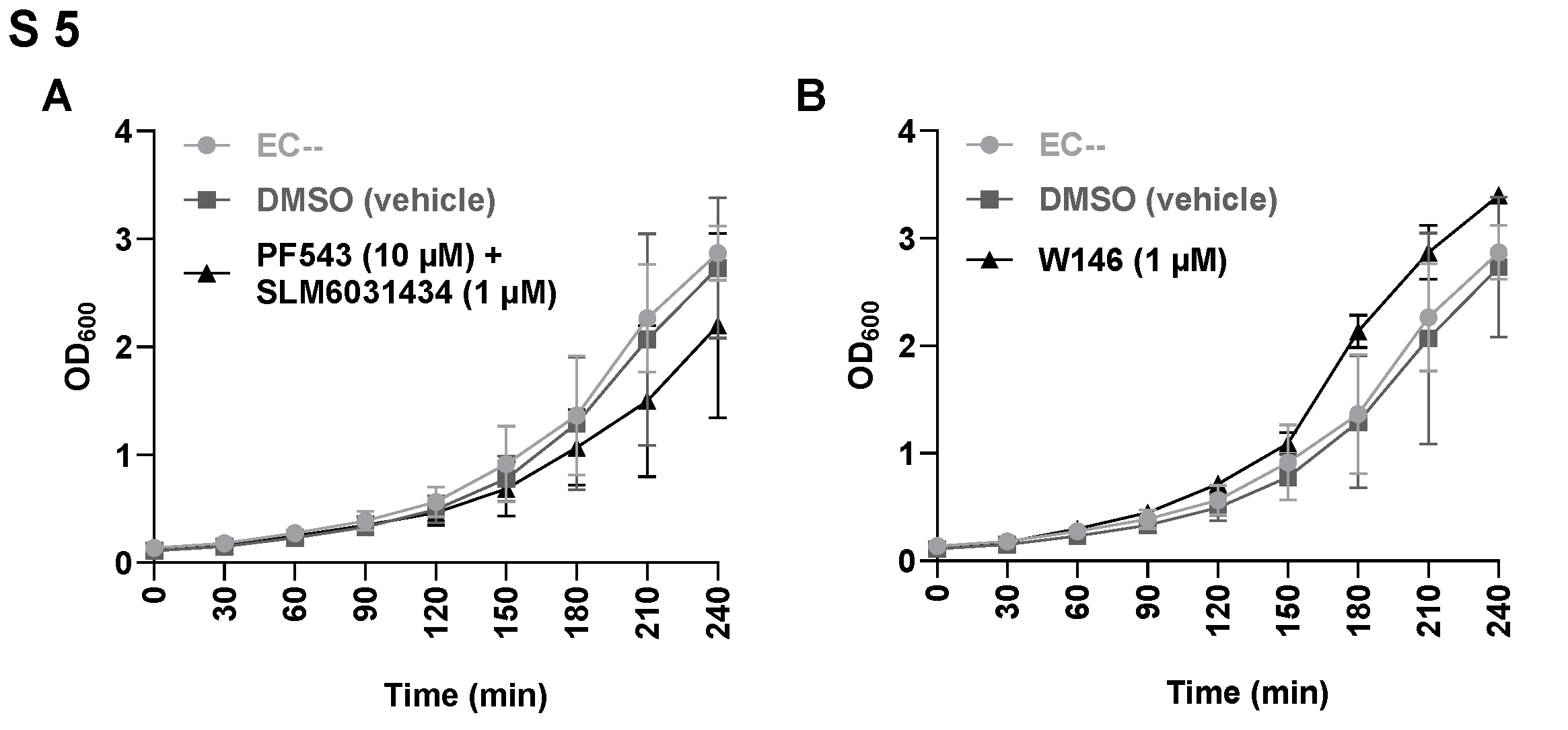

Supplement: Supplementary file 5 — Supplementary Material 5 [file 12987_2025_687_MOESM5_ESM.tif]

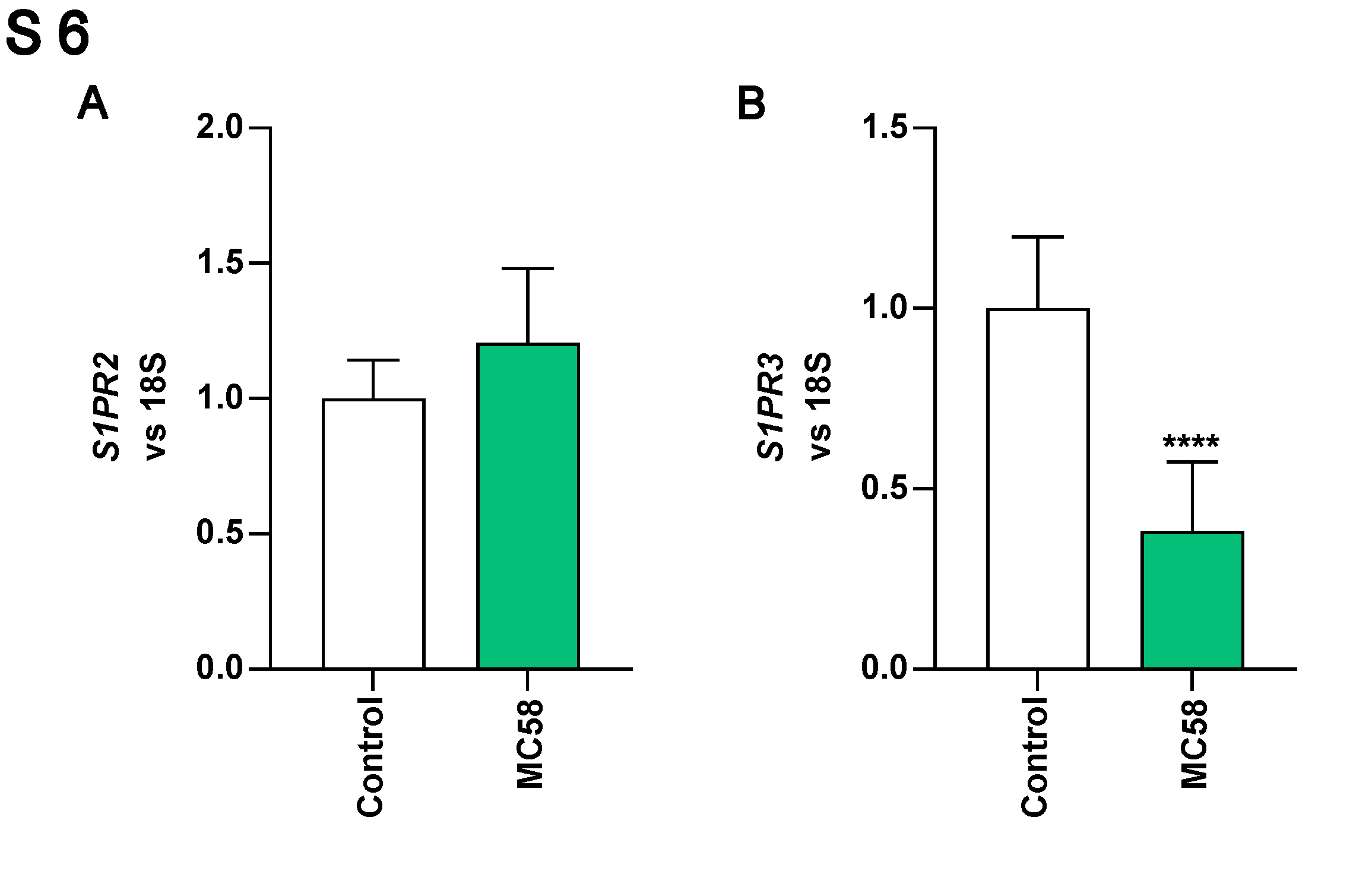

Supplement: Supplementary file 6 — Supplementary Material 6 [file 12987_2025_687_MOESM6_ESM.tif]

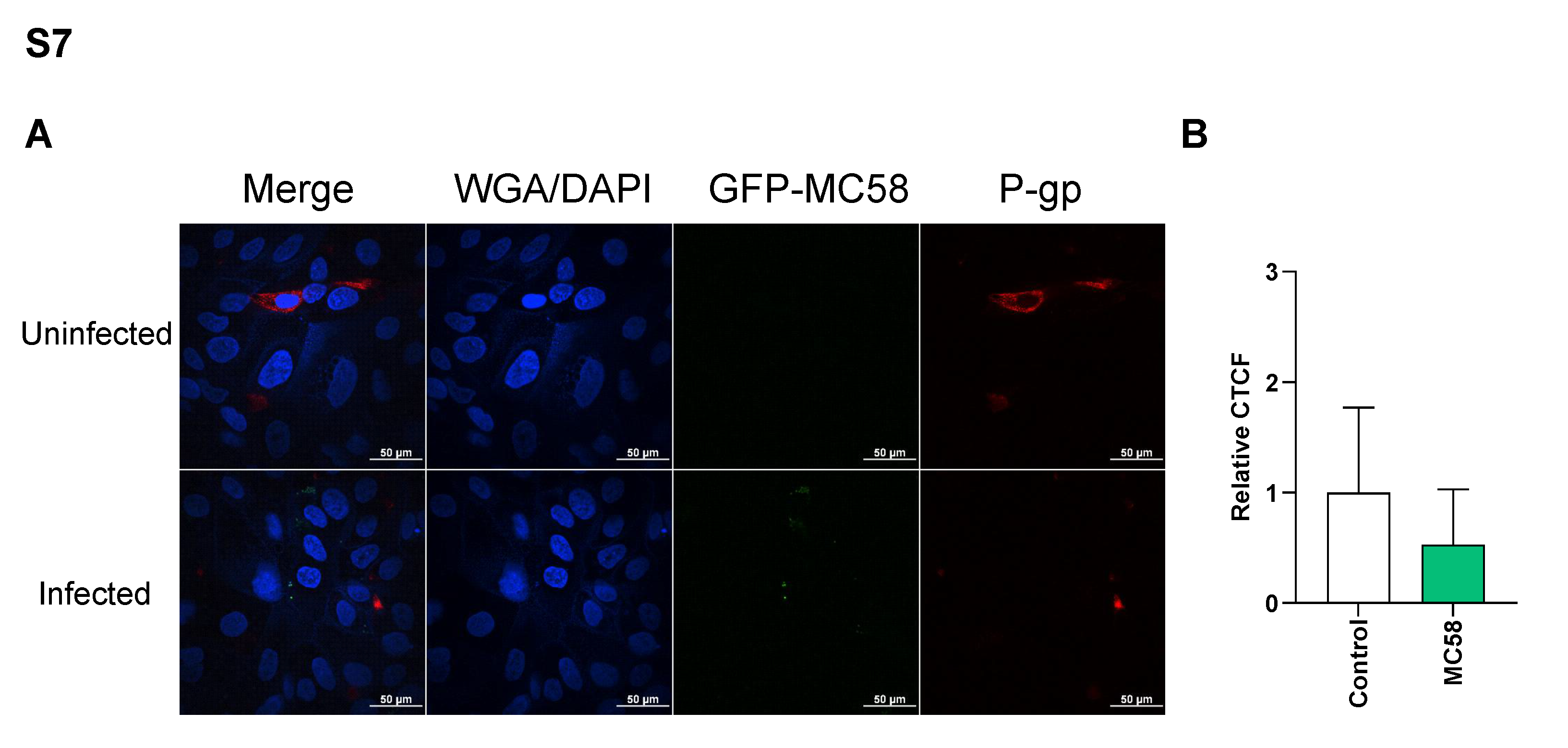

Supplement: Supplementary file 7 — Supplementary Material 7 [file 12987_2025_687_MOESM7_ESM.tif]

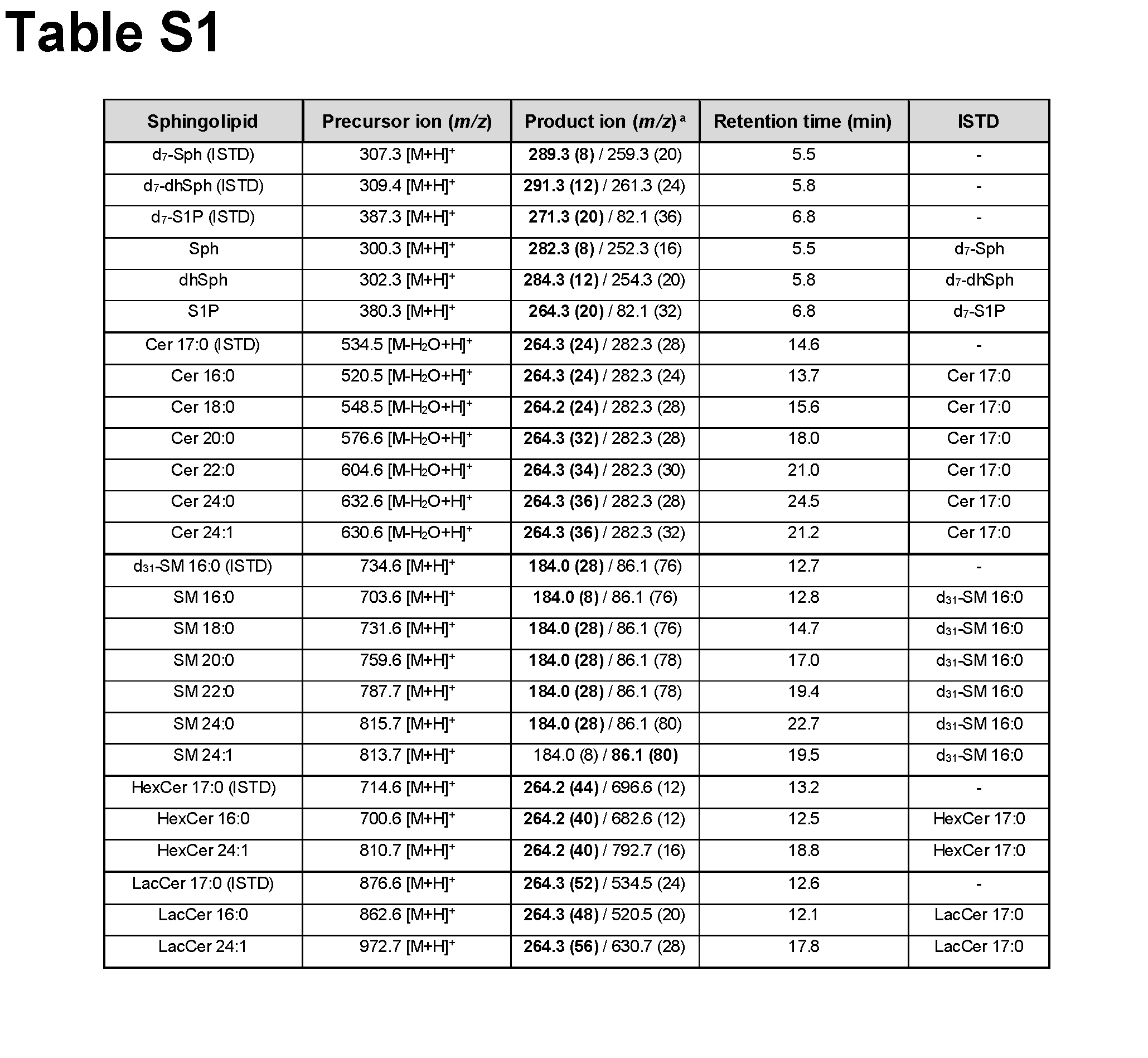

Supplement: Supplementary file 8 — Supplementary Material 8 [file 12987_2025_687_MOESM8_ESM.tif]
